# High‐Performance Sensing Platform Based on Morphology/Lattice Collaborative Control of Femtosecond‐Laser‐Induced MXene‐Composited Graphene

**DOI:** 10.1002/advs.202404889

**Published:** 2024-07-23

**Authors:** Ruige Su, Misheng Liang, Yongjiu Yuan, Chaojun Huang, Wenqiang Xing, Xiaomeng Bian, Yiling Lian, Bo Wang, Zheng You, Rui You

**Affiliations:** ^1^ Laboratory of the Intelligent Microsystem Beijing Information Science and Technology University Beijing 100192 P. R. China; ^2^ School of Instrument Science and Opto‐Electronics Engineering Beijing Information Science and Technology University Beijing 100192 P. R. China; ^3^ Department of Mechanical Engineering City University of Hong Kong Hong Kong 999077 P. R. China; ^4^ School of Mechanical Engineering, Beijing Institute of Technology Beijing 100081 P. R. China; ^5^ Institute of Medical Equipment Science and Engineering Huazhong University of Science and Technology Wuhan 430074 P. R. China; ^6^ State Key Laboratory of Precision Testing Technology and Instruments Tsinghua University Beijing 100084 P. R. China

**Keywords:** high‐performance sensing platform, laser‐induced graphene, morphology/lattice control, MXene‐composited graphene, probe and pump technology

## Abstract

Flexible sensors based on laser‐induced graphene (LIG) are widely used in wearable personal devices, with the morphology and lattice arrangement of LIG the key factors affecting their performance in various applications. In this study, femtosecond‐laser‐induced MXene‐composited graphene (**LIMG**) is used to improve the electrical conductivity of graphene by incorporating MXene, a 2D material with a high concentration of free electrons, into the LIG structure. By combining pump‐probe detection, laser‐induced breakdown spectroscopy (LIBS), and density functional theory (DFT) calculations, the morphogenesis and lattice structuring principles of **LIMG** is explored, with the results indicating that MXene materials are successfully embedded in the graphene lattice, altering both their morphology and electrical properties. The structural sparsity and electrical conductivity of **LIMG** composites (up to 3187 S m^−1^) are significantly enhanced compared to those of LIG. Based on these findings, **LIMG** has been used in wearable electronics. **LIMG** electrodes are used to detect uric acid, with a minimum detection limit of 2.48 µM. Additionally, **LIMG**‐based pressure and bending sensors have been successfully used to monitor human limb movement and pulse. The direct in situ femtosecond laser patterning synthesis of **LIMG** has significant implications for developing flexible wearable electronic sensors.

## Graphical abstract

In this study, femtosecond‐laser‐induced MXene‐composited graphene (**LIMG**) is successfully synthesized. Transmission electron microscopy （**TEM**） analysis shows that the lattice structures of MXene and graphene are interspersed with each other. The study also elucidates the mechanism of **LIMG** enhanced conductivity. **LIMG** is successfully applied to a high‐performance multifunctional sensing platform.

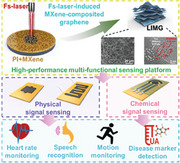

## 1 Introduction

Wearable sensors, typically worn in close proximity to or directly on the skin, are small devices that monitor and collect real‐time data on the physiological and biochemical parameters of the human body^[^ ^1^ ^]^ and thus exhibit substantial potential in remote health monitoring, physical activity tracking, human‐computer interactions, and real‐time physiological signal monitoring.^[^ ^2^ ^]^ The evolution of flexible wearable sensors with multifunctionality and high performance has increased the demand for sensitive materials.^[^ ^3^ ^]^ In scenarios involving the monitoring of multiple physiological signals, it is important to be sensitive to both physical changes, such as pressure and strain, and biochemical signals, such as disease markers and protein molecules.^[^ ^4^ ^]^ Addressing physical stimuli requires modification of the micromorphology and porosity of the material.^[^ ^5^ ^]^ Responding to biochemical signals necessitates improving the electrical properties of the material and integrating specific sensitive substances.^[^ ^6^ ^]^ Therefore, the coordinated regulation and optimization of the morphology of sensitive units and their electrical performance are of great significance for multifunctional wearable sensors.^[^ ^7^ ^]^

In recent years, laser‐induced graphene (LIG) has been widely utilized in flexible sensors owing to its rapid patterning, mask‐free manufacturing, in‐situ synthesis, and cost‐effective production of graphene‐based materials.^[^ ^8^ ^]^ Hence, LIG shows significant promise, particularly for multisignal sensing.^[^ ^9^ ^]^ The 3D porous structure of the LIG provides numerous surface sites for chemical reactions and physical interactions.^[^ ^10^ ^]^ Additionally, LIG interacting with electrolytes, metabolites, hormones, and other chemical markers triggers changes in the potential, current, and resistivity, thus enabling the detection and analysis of analytes based on these alterations.^[^ ^11^ ^]^ In particular, the surface morphology, internal structure, and porosity of LIG can be controlled by adjusting the laser‐processing conditions, offering a straightforward and feasible approach for tuning the micromorphology and electrical properties of LIG‐based sensitive units.^[^ ^12^ ^]^ LIG is used in a variety of sensors, including electrochemical sensors and pressure sensors.^[^ ^13^ ^]^ However, the electrical conductivity and carrier mobility of LIG still require improvement owing to their irregular lattices and discontinuous interlayer structures.^[^ ^14^ ^]^ Researchers have attempted various strategies, such as spin‐coating highly conductive MXene and silver nanowires,^[^ ^15^ ^]^ electrodepositing nanoparticles,^[^ ^16^ ^]^ and secondary reduction of laser‐induced metal precursor solutions,^[^ ^10c^ ^]^ to composite LIG. However, these doping methods, used as secondary post‐production processes, only deposit the doping materials on the surface of LIG without penetrating the lattice, and the relatively unstable binding of LIG introduces additional uncertainties in the material preparation process.^[^ ^17^ ^]^ Previous LIG production methods involved long‐pulse or continuous laser irradiation, which limited the controllability of the micromorphology and patterning resolution owing to the high instability of the polymer films under thermal effects.^[^ ^18^ ^]^

In this study, we incorporated MXene into the polyimide precursor solution to obtain MXene‐mixed polyimide films. Using a femtosecond laser direct writing process, we fabricated porous graphene embedded with MXene lattice. By leveraging the low thermal impact of the femtosecond laser, we successfully fabricated femtosecond‐laser‐induced MXene‐composited graphene **(LIMG)** with a minimum linewidth of 1 µm via direct laser writing on polymer films. This unique precursor doping technique enabled the uniform doping of MXene within the lattice of LIG, creating a stable environment for carrier transport across the defect‐ridden lattice of LIG. Compared to the original LIG, **LIMG** displayed enhanced carrier mobility and significantly improved electrical conductivity, enhanced by two orders of magnitude to 3187 S m^−1^. Additionally, we investigated the impact and control of the femtosecond laser on the micromorphology of **LIMG** using pump‐probe and LIBS techniques. Through the coordinated optimization of lattice doping and micromorphology, we established an **LIMG** material platform with exceptional electrochemical, pressure, and strain‐sensing capabilities. We successfully developed a highly sensitive electrochemical sensor for the detection of uric acid, with a minimum detection limit of 2.48 µM. The pressure sensor demonstrated a broad detection range (0–200 kPa), high sensitivity (0.15 kPa^−1^), and good durability (capable of withstanding 1000 cycle tests), while the bending sensor exhibited an optimal detection range and durability over 1000 cycles. These sensors can monitor various physiological signals such as voice, pulse, finger movement, and knee joint motion, making them promising for human health monitoring and human‐machine interaction applications.

## 2 Results and Discussion

### 2.1 Fabrication and Characterization of the femtosecond‐laser‐induced MXene‐composited graphene (LIMG)

**Figure 1:**
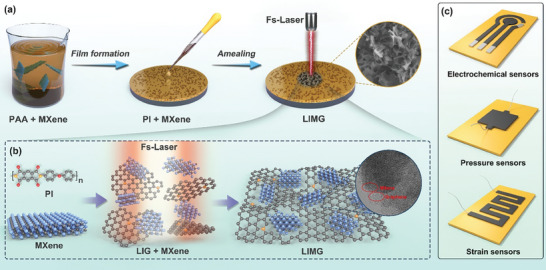
a) Schematic representation of the synthesis of **LIMG** using femtosecond laser ablation. b) Microscopic illustration of **LIMG** prepared by femtosecond laser ablation. c) **LIMG** employed in the fabrication of biosensors, pressure sensors, and bending sensors.

Poly(amido‐amine) (PAA) serves as a precursor to polyimide (PI), which is achieved through coating and high temperature imidization to produce PI materials with superior properties. Incorporating other materials into PAA through physical mixing provides a straightforward method for precursor doping. The precursor material, MXene (Ti_2_CTx), was incorporated into poly(amido‐amine) (PAA) to create PI‐MXene composite films, as shown in **Figures** **1**a and S1 (Supporting Information). Various amounts of MXenes were added to a fixed quantity of PAA and mixed thoroughly to obtain a uniform blend. The resulting composite film was denoted as PI‐MXene‐X (X = 0.3, 0.6, and 0.9 mmol, indicating the MXene loading amount) based on the molar ratio of MXene in PAA. A mixture of MXene and PAA was applied to a clean silicon wafer, where it naturally spread owing to the surface tension. Gradient annealing was then conducted on a hot plate to eliminate solvents, forming a PI‐MXene composite film with a brown texture. The distribution of MXene nanosheets within the composite film is shown in Figure S1d–f (Supporting Information). Increasing doping concentration notably enhanced the internal distribution of MXene nanosheets in the composite film. The setup of the femtosecond‐laser direct‐write processing system is shown in Figure S2 (Supporting Information). **LIMG** was fabricated by irradiating the PI‐MXene composite films with a femtosecond laser in situ.

Figure 1b depicts the interaction between the femtosecond laser and materials. When the femtosecond laser was focused on the PI‐MXene, the material interacted with the laser, resulting in the generation of seed electrons, primarily through field ionization (multiphoton ionization and tunnel ionization) induced by the intense electric field of the femtosecond laser. These excited electrons changing from the bonding to an antibonding state weaken the electronic bonds near the carbon atoms at the top of the valence band, directly leading to the ionization of oxygen and carbon atoms.^[^ ^19^ ^]^ Exposure to a femtosecond laser excited a significant number of free electrons, which gathered around the polyimide and MXene, leading to the breaking of the C─O and C═O bonds in polyimide and Ti bonds in MXene, eventually resulting in the formation of plasmas.^[^ ^20^ ^]^ Subsequently, electron–hole recombination occurs on the surfaces of the polyimide and MXene, creating new materials.^[^ ^21^ ^]^ During this process, some carbon and oxygen atoms are released in the form of CO and CO_2_ gases, which directly contribute to the development of a three‐dimensional porous structure.^[^ ^22^ ^]^ The remaining free carbon atoms realign and combine to form graphene, whereas the recombined MXene particulates are incorporated into the graphene lattice, creating a composite lattice structure comprising graphene and MXene. Leveraging the high‐sensitivity response of MXenes to biological molecules, combined with the conductivity, porosity, flexibility, and biocompatibility of graphene,^[^ ^23^ ^]^ enables the development of high‐performance sensors and detection devices suitable for wearable electronic applications. The application of **LIMG** in producing flexible wearable electronic devices such as biosensors, pressure sensors, and bending sensors is illustrated in Figure 1c.

**Figure 2:**
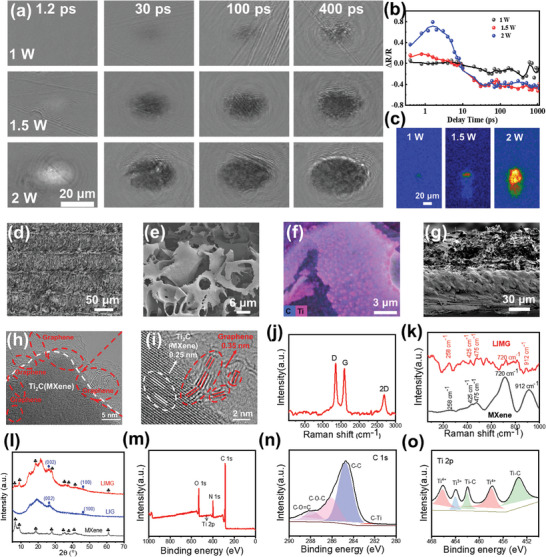
a) Spatiotemporal evolution process of the relative reflectivity on the surface of MXene (MXene mixing concentration: 0.3 mmol, under the excitation of a fluence ranging from 1 to 2 W). b) Actual measured relative reflectance change. c) Plasma luminescence intensity acquired by ICCD at a delay of 5 ns. d, e) Scanning electron microscopy (SEM) images of the surface of LIMG (MXene concentration of 0.6 mmol, laser repetition frequency 500 kHz, laser power 2 W, scanning speed 5 mm^−1^ s). f) In the lamellar structure of LIMG, the elemental mapping images depict carbon elements (colored red) and titanium elements (colored green). g) Cross‐sectional SEM image of LIMG (MXene concentration of 0.6 mmol, laser repetition frequency 500 kHz, laser power 2 W, scanning speed 5 mm^−1 ^s). h) Transmission electron microscopy (TEM) image of LIMG (MXene concentration of 0.6 mmol, laser repetition frequency 500 kHz, laser power 2 W, scanning speed 5 mm^−1^ s). i) High‐resolution TEM (HRTEM) image depicting the lattice spacing of LIMG. j) Raman spectroscopy of LIMG (MXene concentration of 0.6 mmol, laser repetition frequency 500 kHz, laser power 2 W, scanning speed 5 mm^−1^ s). k) Raman spectra of LIMG and MXene at low wave numbers. l) X‐ray diffraction (XRD) patterns of LIMG (MXene concentration of 0.6 mmol, laser repetition frequency 500 kHz, laser power 2 W, scanning speed 5 mm^−1^ s). m, n, o) X‐ray photoelectron spectroscopy (XPS) of C 1s and Ti 2p for LIMG (MXene concentration of 0.6 mmol, laser repetition frequency 500 kHz, laser power 2 W, scanning speed 5 mm^−1^ s).

This study utilized a pump‐probe approach combined with LIBS to investigate the multi‐scale observations of lattice melting, surface fragmentation and plasma eruption of polyimide under femtosecond laser irradiation, as illustrated in **Figures** **2**a,b and S3 (Supporting Information). Pump‐probe detection revealed differences in the surface spallation phenomena and thermal expansion at various power levels—possibly key factors influencing the surface morphology of **LIMG**. Furthermore, the plasma ejection intensities at different powers indicated that the plasma ejection at 2 W was significantly higher than that at 1 W. At a low flux, minor ablation occurs when the material is vaporized, often resulting in a smaller decrease in reflectivity, and the introduced defects and oxidation density are low. With increased flux, thermal effects can cause severe damage to the material, with a significant number of chemical bonds breaking, material decomposition, and thermal stress‐inducing internal strain in the material, thereby altering its morphology and properties. As per the plasma ejection intensity captured by the ICCD at a delay of 5 ns (Figure 2c), substantial differences are observed in the instantaneous temperatures and thermal effects. The plasma ejection intensity at 1.5 W was slightly higher than that at 1 W. However, at 2 W, the intensity of the plasma ejection was significantly greater. Notably, Figure S3b,c (Supporting Information) demonstrate that at the same laser fluence, a higher doping concentration resulted in a more pronounced increase in reflectivity between 1–2 ps.

Figure 2d,e show the scanning electron microscope images of the prepared **LIMG**. Under a laser power of 2 W, the **LIMG** formed possesses a larger specific surface area and a porous structure, with three‐dimensional porosity clearly observable under higher magnification. Notably, the porous nanosheets of **LIMG** have an ultrathin randomly stacked network setup, resulting from the rapid release of gases produced during the thermolysis and carbonation procedures, owing to the breakdown of C─O, C═O, and C─N bonds in the polymer film.^[^ ^24^ ^]^ When the temperature decreased, a porous structure formed on the surface of the polymer. Figure S4 (Supporting Information) shows the micromorphology of **LIMG** under various laser powers. At a laser power of 500 mW, slight indentations were observed on the surface of the PI‐MXene film. With increasing power, ablation occurred progressively, revealing different micromorphologies, consistent with the results observed in the pump‐probe measurements. At a laser power of 2 W, the **LIMG** surface exhibits a lamellar structure with high porosity. As shown in Figure S4d (Supporting Information), scanning the laser a second time disrupted the microstructure on the surface of the **LIMG**. The elemental mapping images of **LIMG** (Figure 2f) illustrate the distribution of carbon and titanium within the fabricated layered structures. Figure S5 (Supporting Information) presents the energy‐dispersive spectroscopy (EDS) spectrum scan, further elucidating the relative distribution of titanium and carbon. This setup offers numerous sites for adsorption and plays a vital role in providing efficient carrier transmission and rapid adsorption and desorption.^[^ ^25^ ^]^ The cross‐sectional SEM image in Figure 2g illustrates the **LIMG** structure with a laser scribing depth of approximately 45 µm. The porous nature of **LIMG**, which resembled a loose porous structure with sponge‐like compositions, was further confirmed by the SEM cross‐section. Additionally, Figure S4 (Supporting Information) shows the surface morphology of **LIMG** at different fabrication powers, showing an increase in the porous structure at higher laser powers. When the surface was subjected to secondary processing with a 2 W laser power, the microstructures disappeared owing to excessive ablation. As depicted in Figure S6a (Supporting Information), the minimum feature size of **LIMG** produced by femtosecond laser machining can be refined to approximately 1 µm under a 100X objective lens, representing a significant enhancement in processing precision compared with CO_2_ lasers. As demonstrated in Figure S6b (Supporting Information), the direct laser writing of ultra‐micro interdigitated electrodes can be easily achieved.

The transmission electron microscopy (TEM) results of **LIMG** (Figure 2 h,i; Figure S7, Supporting Information) revealed that **LIMG** comprised interconnected graphene nanoparticles, with MXene nanoparticles dispersed among them, as shown in Figure S7a (Supporting Information). Additionally, Figure S7b (Supporting Information) shows the mutual embedding of MXene nanoparticles and graphene. Figure S7c (Supporting Information) provides a clearer view of the mutual embedding and composite nature of the MXene lattice with the graphene lattice, with distinct lattice stripes of meandering graphene visible under the regions containing the MXene lattice. Figure 2h shows the interwoven lattice structures of the graphene and MXene. The MXene lattice fills the structural defects in graphene, enhancing the density of free electrons and improving the conductive interface of the entire **LIMG**.^[^ ^26^ ^]^ As shown in Figure 2i, the high‐resolution TEM (HRTEM) image of **LIMG** displays several lattice fringes at different distances. These fringes are related to both graphene and MXene, where the 0.35 nm lattice spacing corresponds to the (002) crystal plane of graphene, while the 0.25 nm lattice spacing corresponds to the (004) crystal plane of MXene.

**LIMG** was further characterized using Raman spectroscopy to study its phase composition and valence bonds. The Raman spectrum of **LIMG** (Figure 2j,k) exhibits two characteristic peaks at 1351 and 1588 cm^−1^, corresponding to the D and G bands, respectively, indicating typical carbon characteristics. The Raman wavenumber of 2660 cm^−1^ (2D peak) and the ratio of the intensities of the G and D peaks (IG/ID = 1.04) signify the formation of highly multilayered graphene, consistent with the X‐ray diffraction (XRD) results.^[^ ^27^ ^]^ Figure 2k shows that the characteristic peaks of **LIMG** at lower wavenumbers coincide with those of MXene, indicating the presence of MXene within the graphene. Figure S8 (Supporting Information) shows the Raman spectra of **LIMG** at varying power levels, with the crystallinity of graphene peaking at a power of 2 W. The XRD pattern of **LIMG** (Figure 2l) demonstrates the highly crystalline structure of the graphene material; the intense peak (45.1°) indicates the (100) reflection in graphene, while the asymmetric peak (002) in the 20–30° range signifies the existence of an amorphous structure in **LIMG**. The other characteristic peaks were largely consistent with the peak positions of the original MXene, further confirming the formation of a composite of graphene and MXene. X‐ray photoelectron spectroscopy (XPS) characterization was performed to further ascertain the chemical state and composition of **LIMG**. The XPS spectra (Figure 2m) revealed the presence of Ti, C, O, and N in the sample. The high‐resolution C 1s and Ti 2p XPS spectra are shown in Figure 2n,o, respectively. The C1s signal was deconvoluted into four peaks located at 281.5, 284.9, 286.7, and 288.7 eV, corresponding to C─Ti, C═C, C─O, and O─C═O, respectively. The Ti 2p spectrum further confirms the presence of Ti‐C bonds.

### 2.2 Conductivity Studies of the LIMG

**Figure 3:**
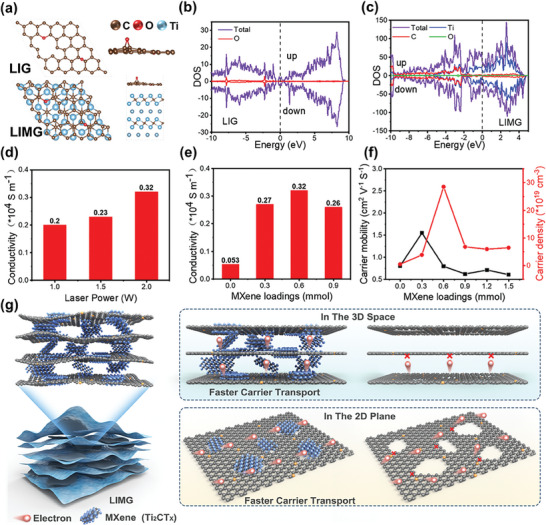
a) Schematic diagram of the lattice modeling for LIG and **LIMG**. b) Density of states (DOS) of LIG. c) DOS of **LIMG**. d) Conductivity of **LIMG** under varying laser power levels. e) Conductivity of **LIMG** with different concentrations of MXene doping. f) Carrier mobility (black line) and carrier density (red line) of **LIMG** at various MXene doping concentrations. g) Schematic diagram depicting the mechanism of conductivity enhancement in **LIMG**.

Density functional theory (DFT) calculations were performed using projector‐augmented wave (PAW) pseudopotentials in conjunction with Perdew–(Burke)–Ernzerhof functions,^[^ ^28^ ^]^ as implemented in the Vienna ab initio simulation package (VASP) code.^[^ ^29^ ^]^ An energy cutoff of 450 eV was used for the plane‐wave basis expansion. LIG and **LIMG** models were constructed, as shown in **Figure** **3**a. The unit cell of graphene/MXene heterostructures was constructed by placing a 5 × 5 graphene sheet over a 4 × 4 MXene supercell, along with a tiny lattice mismatch of 0.8%; in addition, a vacuum layer of 15 Å was adopted to avoid the spurious image interaction. The unit cell was adopted for graphene/MXene structural relaxation and electronic property calculations, with the Brillouin zone integration sampled by 2 × 2 × 1 and 5 × 5 × 1 k‐grid mesh,^[^ ^30^ ^]^ respectively. Full relaxation of the atomic positions, including the shape and size of the supercell, was achieved by employing the conjugate gradient method, with the convergence criteria set at 10^−5^ eV and −0.02 eV/Å for energy and force, respectively. Long‐range van der Waals (vdW) interactions were considered using the DFT‐D3 method.^[^ ^31^ ^]^ As shown in Figure 3a, we introduced oxygen defects into the graphene lattice to better reflect the actual LIG structure. The density of states (DOS) results in Figure 3b,c show that both LIG and **LIMG** exhibit asymmetric magnetic characteristics in the spin‐up and spin‐down states. Notably, compared to LIG, **LIMG** displays a higher density of states near the Fermi level, which may enhance its conductivity. The significant increase in the DOS at the Fermi level for **LIMG** is mainly attributed to the contribution of titanium atoms. This indicates that the incorporation of titanium atoms into the lattice significantly increases the electron density, thereby improving the material's conductivity.

We conducted a detailed study of the electrical properties of **LIMG**, with the effects of various processing conditions on **LIMG** conductivity explored. Tables S1,S2, and S3 (Supporting Information) present the conductivity test results. Figure 3d compares the optimal conductivity results of **LIMG** at different laser powers. As the laser power increases, the conductivity steadily increases. A threshold laser power of 2 W is used. Beyond this limit, excessive laser ablation degraded the quality of graphene formation (Figure S8, Supporting Information). Figure S9 (Supporting Information) shows the effect of the laser repetition frequency on the conductivity of the material. With the selected repetition frequencies of 100, 500, and 1000 kHz, the optimal laser repetition frequency was found to be 500 kHz. Low thermal effects at lower repetition frequencies are unfavorable for graphene formation, whereas excessive thermal ablation at higher frequencies negatively affects graphene formation. Figure S10 (Supporting Information) shows a comparison of the optimal conductivity results for **LIMG** at different processing speeds. The conductivity decreases with increasing scanning speed, with an optimal processing speed of 5 mm^−1^s.

Figure 3e shows the conductivity of **LIMG** under optimal processing conditions with different amounts of MXene doping. Compared with pure LIG, adding MXene significantly improved the conductivity of LIG. This increasing trend continues until the concentration of MXene reaches 0.6 mmol, at which point the conductivity peaks at 3187 S m^−1^. As shown in Figure 3f, the carrier mobility and density of **LIMG** with different MXene doping concentrations were tested. Owing to the defective lattice of LIG, the carrier mobility is relatively low. At a doping concentration of 0.3 mmol, **LIMG** exhibits the highest carrier mobility. As the doping concentration increased further, the carrier mobility of **LIMG** exhibited a downward trend, becoming slightly lower than that of LIG, possibly suggesting that introducing excess MXene causes more defects in the system. Owing to the introduction of Ti atoms, the carrier density in the system reached its optimum value at a doping concentration of 0.6 mmol. As the doping concentration continued to increase, the carrier density decreased, further confirming that introducing excessive MXene was detrimental to the material system. As the change in carrier mobility is relatively small, **LIMG** with a doping concentration of 0.6 mmol has the highest carrier density, thus exhibiting the best conductivity at this doping level.

Figure 3g examines the enhanced conductivity mechanism of **LIMG** after MXene incorporation. The exceptional conductivity of MXenes can modify the atomic arrangement and charge distribution within the lattice structure when combined with LIG, ultimately altering the electronic structure of the material. This modification is advantageous for regulating electron transport and provides new possibilities for electronic device design and optimization.^[^ ^32^ ^]^ During the femtosecond‐laser‐induced fabrication process of **LIMG**, the high temperature produced by the laser converts the PI polymer into graphene. However, lattice defects in LIG can diminish its conductivity, thereby limiting its applications.^[^ ^33^ ^]^ The analysis in Figure 2i reveals that when PI‐MXene films generate graphene under the influence of femtosecond lasers, the MXene particles are fragmented by the laser and integrated into the graphene lattice defects. The thinned, reduced, and fragmented MXene particles under the influence of the femtosecond laser supplied a significant number of free electrons at the MXene‐LIG interface, effectively filling the inherent lattice defects of LIG. This defect‐filling process promotes smoother electron transfer across the lattice plane than in LIG. Moreover, the insertion of MXene between the lattice planes creates additional pathways for interlayer electron transfer, enhancing electron transfer on both two‐dimensional and three‐dimensional scales.^[^ ^34^ ^]^ MXenes serve as effective bridges for electron transfer.^[^ ^35^ ^]^ Therefore, we selected a 0.6 mmol MXene optimal doping ratio with the best laser processing conditions—laser repetition frequency: 500 kHz; laser power: 2 W; scanning speed: 5 mm ^−1^s—to conduct further application research.

### 2.3 Electrochemical Performance Evaluations and Uric Acid Sensing

**Figure 4:**
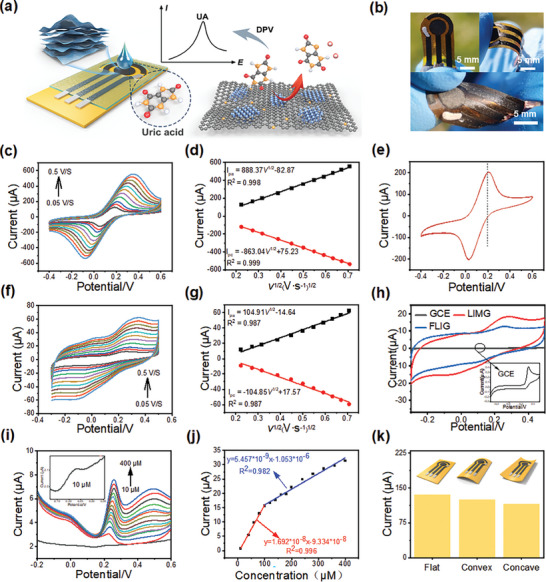
a) Schematic diagram of the working principle of electrochemical sensors. b) Photographs of the flexible electrochemical chip at various bending angles. c) Cyclic voltammetry (CV) measurements conducted at different scan rates (0.05, 0.75, 0.1, 0.15, 0.2, 0.25, 0.3, 0.35, 0.4, 0.45, and 0.5 V·s^−1^) of the **LIMG**‐based electrode in 5 mM K_3_[Fe(CN)_6_]/K_4_[Fe(CN)_6_] and 0.1 M KCl. d) Peak current plotted as a function of the square root of the scan rate with fitted linear regression curves in 5 mM K_3_[Fe(CN)_6_]/K_4_[Fe(CN)_6_] and 0.1 M KCl. Black and red dots indicate anodic and cathodic peak currents, respectively. e) 10 cycles of CV recorded at a scan rate of 0.1 V·s^−1^ in 5 mM K_3_[Fe(CN)_6_]/K_4_[Fe(CN)_6_] and 0.1 M KCl. f) CV measurements conducted at different scan rates (0.05, 0.75, 0.1, 0.15, 0.2, 0.25, 0.3, 0.35, 0.4, 0.45, and 0.5 V·s^−1^) of the **LIMG**‐based electrode in 50 µM UA and 0.01 M PBS. g) Peak current plotted as a function of the square root of the scan rate with fitted linear regression curves in 50 µM UA and 0.01 M PBS. Black and red dots indicate anodic and cathodic peak currents, respectively. h) Comparison of CV curves of **LIMG**‐based and glassy carbon electrodes in 50 µM UA and 0.01 M PBS (inset: CV curve of glassy carbon electrode). i) DPV curves measured with different concentrations of UA using the **LIMG**‐based electrode. j) Fitted calibration curve of the **LIMG**‐based electrode between peak currents and UA concentrations. k) Peak currents for different bending states of the sensor in 5 mM K_3_[Fe(CN)_6_]/K_4_[Fe(CN)_6_] and 0.1 M KCl.

As depicted in Figure S11 (Supporting Information), a co‐planar triple‐electrode system (with working electrode: WE; counter electrode: CE; reference electrode: RE) was fabricated on a PI‐MXene composite film utilizing laser direct writing technology. The reference electrode was constructed via silver paste application.^[^ ^36^ ^]^ Selective passivation with polydimethylsiloxane (PDMS) isolates the sensor region containing active components. To facilitate connection with the electrochemical workstation, copper wires were extended from the ends of the electrodes. **Figure** **4**a presents a schematic illustrating the sensing principle of an electrochemical sensor based on **LIMG**. Within the interface incorporating the sensitive materials graphene and MXene, the uric acid molecule undergoes oxidation and electron loss, resulting in modification of the **LIMG** potential, as confirmed by differential pulse voltammetry. A physical drawing of the electrochemical sensor is shown in Figure 4b, illustrating its flexibility to be bent and twisted as needed.

To study the electrochemical properties of the fabricated **LIMG**‐based three‐electrode system, cyclic voltammetry (CV) curves of this system were measured in a solution of 5 mM K_3_[Fe(CN)_6_]/K_4_[Fe(CN)_6_] and 0.1 M KCl at different scan rates (0.05–0.5 V•s^−1^, as shown in Figure 4c). As shown in Figure 4c, good linear relationships were observed between the peak currents of anodic (Ipa) and cathodic (Ipc) with the square root of scan rate (v^1/2^), which indicates the redox reaction of [Fe(CN)_6_]^3‐/4−^ on the **LIMG** working electrode involves a diffusion process described by Randles–Sevcik equation.^[^ ^37^ ^]^ The linear regression equations are I_pa_ (µA) = −82.87 + 888.37v^1/2^ (V·s^−1^) (R^2^ = 0.998) and I_pc_ (µA) = 75.23 – 863.04v^1/2^ (V·s^−1^) (R^2^ = 0.999), respectively. These equations suggest that effective electron transfer occurs in **LIMG**‐based three‐electrode systems.^[^ ^38^ ^]^ We prepared femtosecond laser induced graphene(FLIG) electrodes without MXene mixing using the same preparation process. As shown in Figure S12 (Supporting Information), FLIG's Ipa and Ipc exhibit a good linear relationship with the square root of the scan rate (v^1/2^). Despite effective electron transfer, the current peaks at various scan rates are significantly lower than those of the **LIMG** electrode. Ten cycles of CV scans were conducted at a scan rate of 0.1 V•s^−1^ to evaluate the performance of the reference electrode in the **LIMG** three‐electrode system. Figure S13 (Supporting Information) compares the CV curves of eight electrodes in 5 mM K_3_[Fe(CN)_6_]/K_4_[Fe(CN)_6_] and 0.1 M KCl. Due to the high stability of the femtosecond laser direct writing process, the CV curves of these sensors are very similar, indicating reliable repeatability. Figures 4e and S14 (Supporting Information) shows that the peak potentials of the anodic (Epa) and cathodic (Epc) electrodes remained consistent after ten scans, indicating that the silver reference electrode offered a stable reference potential.

This study investigated the possibility of quantitatively analyzing the uric acid (UA) using electrodes based on **LIMG**. Uric acid, a significant metabolic by‐product of the human body found in sweat and urine, requires accurate measurement for diagnostic purposes owing to its association with various diseases when its levels are high. The electronic transfer of UA onto the **LIMG** electrodes was examined, as shown in Figure 4f. Owing to the inherent electroactivity of uric acid, no additional modification of the working electrode was necessary. CV measurements of 50 µM UA in 0.01 M PBS demonstrated the relationship between peak current and the square root of scan rate, indicating that the redox process of UA at the **LIMG** working electrode is diffusion‐limited. The deviation from linearity and the non‐Nernstian response at slower scan rates are attributed to quasi‐reversibility and the possibility of irreversible chemical reactions.^[^ ^39^ ^]^ Figure 4g demonstrates a ΔEp of 210 mV at a scan rate of 0.05 V·s^−1^, showing that ΔEp changes with different scan rates, providing additional evidence for a quasi‐reversible electrochemical process. As shown in Figure S15 (Supporting Information), the properties of the FLIG electrode are similar to those of the **LIMG** electrode. However, CV measurements of 100 µM UA in 0.01 M PBS indicate that the peak current of the FLIG electrode is lower than that of the **LIMG** electrode, despite the higher concentration of UA. Additionally, Figure 4h compares the performance of the **LIMG** electrode, FLIG electrode and commercial glassy carbon electrode in 50 µM UA (0.01 M PBS), indicating that the current response of the **LIMG** electrode to UA is significantly superior to that of the FLIG and glassy carbon electrodes. The CV results demonstrate that the use of the **LIMG** electrode can achieve faster electron transfer. Figure S16 (Supporting Information) shows the long‐term stability test of **LIMG** electrode. In 5 mM K_3_[Fe(CN)_6_]/K_4_[Fe(CN)_6_] and 0.1 M KCl, the peak oxidation current intensity of **LIMG** electrode can still maintain 94% of the original after 20 days, which indicates that **LIMG** electrode has good long‐term stability.

Figure 4i,j illustrate the detection sensitivity and limit of detection (LOD) values for UA using the differential pulse voltammetry (DPV) method, with a linear response range of 10–400 µM determined via linear fitting. As depicted in Figures 4i and S17 (Supporting Information), the LOD for UA is calculated based on the standard deviation (SD) of the response curve fitted at 10 µM and the slope (S) using the formula LOD = 3 × (SD/S). The detection limit for UA was 2.48 µM. The **LIMG**‐based electrodes produced in this study displayed a satisfactory detection range and relatively low LOD. Figure S18 (Supporting Information) shows the DPV curves for detecting UA using the FLIG electrode. It can be observed that the DPV curves at concentrations of 10–20 µM are almost identical, indicating the absence of detectable UA. At a concentration of 50 µM, UA is detectable, and as the concentration of UA increases, the current intensity exhibits a gradient change. Compared to the FLIG electrode, the **LIMG** electrode shows enhanced current intensity and improved detection capability. As shown in Figure S19 (Supporting Information), in a mixed PBS solution of 100 µM AA, 100 µM DA and 100 µM UA, signals appeared at different potentials. This indicates that the **LIMG** electrode has selectivity and potential for sensing multiple substances. As shown in Figure 4k, the electrochemical performance of the electrodes is not significantly affected by torsional deformation, as deduced from the nearly unchanged peak current, thus indicating the remarkable flexibility and stability of the electrodes. This exceptional flexibility and stability render the **LIMG**‐based electrodes advantageous for potential applications in wearable devices.

### 2.4 Pressure and Bending Sensing

Pressure and bending sensors were fabricated using direct laser writing and patterning, as shown in Figures S20 and S21 (Supporting Information), respectively. The manufacturing patterns of the two devices were pre‐drawn and introduced into the laser processing system to draw laser lines. Subsequently, a silver paste was applied to both ends of the electrodes, the copper wires were extended, and the device surface was covered with PDMS. The sensors were baked on a 65 °C hot plate for 3 h. By utilizing the advantages of the laser‐engraving process, sensors can be produced in arrays based on customer‐specified patterns and then cut into small independent units, enabling cost‐effective mass production. The electromechanical characteristics of the pressure sensors were also investigated.

**Figure 5:**
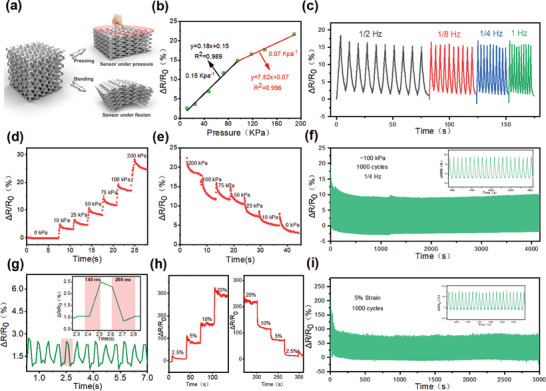
Electromechanical characterization tests of pressure and bending sensors based on **LIMG**, where the pressure sensor has a sensing unit size of 1 × 1 cm^2^, and the bending sensor adopts a serpentine structure. a) Sensing mechanism of the **LIMG**‐based pressure sensor. b) Relative change in resistance of the pressure sensor under external pressure. c) Relative change in resistance during cyclic compression/release at different frequencies (1/8 Hz, 1/4 Hz, 1/2 Hz, and 1 Hz). d, e) Relative change in resistance during stepwise pressurization and depressurization. f) Performance of the pressure sensor under repeated pressure impact tests at ≈100 kPa (over 1000 cycles). g) Response and recovery time of strain sensor with 2.5% bending amount. h) Change in resistance of the **LIMG**‐based bending sensor under different bend amounts. i) Fatigue test of the strain sensor with a bending amount of 5% (over 1000 cycles).

As shown in **Figure** **5**a, the resistance of **LIMG** changes gradually with increasing pressure, attributed to the higher density resulting from the pressure application and the expanded contact areas between the graphene sheets in the 3D porous structure. This behavior resembles that of a compressed sponge, which ultimately enhances conductivity. A 1 × 1 cm solid square pressure sensor based on **LIMG** was analyzed, as depicted in Figure 5b, to investigate the electromechanical mechanism of the pressure sensors. The **LIMG** sensor exhibited high sensitivity, excellent linearity, and a broad detection range. The correlation between the resistance shift on the **LIMG** sensor surface and external pressure (P) is illustrated in the characteristic curve, which shows a positive resistance shift, good linearity, and sensitivity across a detection range of up to 200 kPa. The sensitivity was quantified as follows:

(1) $$s=\frac{\Delta R}{R_{0}\Delta P}\;\times 100\%\;=\frac{R_{RT}-R_{0}}{R_{0}\Delta P}\;\times 100\%$$

where R_0_ represents the initial resistance, ΔR signifies the difference between the real‐time resistance value R_RT_ and initial value R_0_ and ΔP indicates the amount of pressure change. The sensitivity of the sensor was calculated to be 0.15 kPa^−1^ in the range of 0–100 kPa and 0.07 kPa^−1^ in the range of 100–200 kPa. Figure S22 (Supporting Information) shows the performance of the FLIG pressure sensor, which was calculated to have a sensitivity of 0.04 kPa^−1^ in the range of 0–90 kPa and 0.02 kPa^−1^ in the range of 90–200 kPa. The increase in **LIMG** conductivity is favorable for pressure sensing.

The frequency response of the **LIMG** pressure transducer was tested, as shown in Figure 5c. The wide range of frequency responses, from 1/8 to 1 Hz, suggests its ability to function reliably across various pressurization frequencies. Figure 5d,e show the relative resistance change during step‐pressure loading and unloading, respectively, confirming the stability of the sensor under varying pressures. A series of over 1000 mechanical fatigue tests were conducted on the pressure sensor at 100 kPa. Figure 5f shows the durability and stability of flexible **LIMG** pressure sensors. The characteristics of the flexible **LIMG** bending sensors were investigated via bending tests. As shown in Figure 5g, when the bending amount is 2.5%, the response time of the strain sensor is 140 ms and the recovery time is 200 ms. Variations in the resistance at different bending rates highlight the broad detection range of the **LIMG**‐based bending sensor (Figure 5h). Additionally, the strain sensor demonstrated excellent repeatability over more than 1000 bending tests (Figure 5i). The **LIMG** flexible strain sensor exhibited superior linearity, sensitivity, and repeatability when subjected to bending and extrusion.

Table S4 (Supporting Information) compares **LIMG** and LIG piezoresistive sensors with similar device structures. Through material compounding and structural regulation, the **LIMG**‐based pressure sensors exhibit superior performance. Table S5 (Supporting Information) compares the performance of uric acid sensors based on **LIMG** and other graphene‐based composites. In previous studies, to enhance the detection capability of uric acid sensors, various combined processes were often employed to re‐modify the electrode surface with sensitive materials such as metal elements. However, the **LIMG** electrode underwent no secondary processing. It can be observed that the performance of the uric acid sensor based on **LIMG** surpasses that of graphene‐based sensors without secondary modification. Compared to modified sensors, the performance of the **LIMG** electrode is comparable. Notably, the **LIMG** electrode requires only a one‐step preparation process and enables high‐precision manufacturing.

### 2.5 High‐Performance Multi‐Functional Sensing Platform

**Figure 6:**
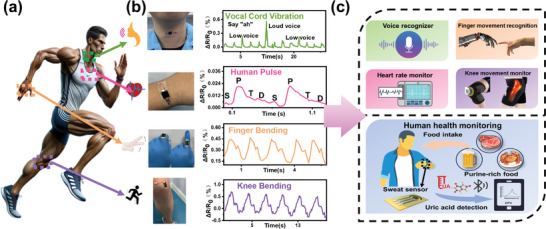
Applications of **LIMG**‐based pressure and strain sensors in detecting human physiological signals: a) identifying human vocal cord phonation; b) monitoring finger movements; c) measuring human pulse; d) assessing knee movements.

**LIMG**‐based sensors offer significant flexibility, high sensitivity, and wide detection ranges for monitoring various physiological signals and body movements. The fabricated flexible devices illustrated in **Figure** **6**a were positioned on vocal cords, pulse points, fingers, and knees to monitor human physiological activity. In Figure 6b, the **LIMG**‐based sensor outputs different electrical signals corresponding to various physiological activities, demonstrating its ability to distinguish between high and low tones. The flexible device can be easily affixed to the wrist using an adhesive tape for pulse measurement. Furthermore, **LIMG**‐based sensors can accurately capture subtle pulse signals, such as p‐, t‐, and d‐waves. When attached to a finger, these sensors can provide real‐time information on their state. Similarly, by attaching an **LIMG**‐based sensor to the knee, knee motion can be detected, indicating its potential applications in exercise recovery. As shown in Figure 6c, these findings may contribute to the future fabrication of sensors for speech recognition, health monitoring, human‐computer interaction, and sports rehabilitation. In conclusion, given their excellent properties, **LIMG**‐based sensors are promising candidates for multifunctional applications in wearable human sensors.

## 3 Conclusion

This paper describes a method for producing laser‐induced MXene‐composited graphene using femtosecond laser manufacturing technology. By combining pump‐probe spectroscopy with LIBS observations and DFT calculations, we investigated the morphological formation process and lattice formation patterns of **LIMG**. We incorporated MXenes into a graphene lattice and manipulated its morphology. Transmission electron microscopy revealed that the lattices of MXene and graphene were interwoven, and introducing MXene significantly improved the electrical conductivity of LIG. Under optimal laser conditions, **LIMG** exhibited an excellent conductivity of 3187 S m^−1^ and exceptional chemical reliability. An electrochemical sensor designed to detect uric acid was prepared using a laser direct‐writing patterning process under optimal processing conditions. This sensor has an excellent detection range with a minimum detection limit of 2.48 µM. Finally, pressure and bend sensors used to detect human physical signals were fabricated based on **LIMG**. These sensors possess good detection range and durability and have successfully demonstrated their immense potential in monitoring various body movements and voice recognition. Overall, the femtosecond laser direct‐write patterning preparation of **LIMG** offers the potential to introduce convenient, high‐performance, and efficient flexible wearable sensors in everyday life.

## 4 Experimental Section

### Preparation of PI/MXene Films

MXene nanosheets (Ti_2_CT_X_) were obtained from Xianfeng Nano Material Company. The MXene sheet diameters were 2–15 µm, and the thicknesses were 100 nm‐1 µm. Poly(amido‐amine) (PAA) solution (type 217) was obtained from Changzhou Ya'an New Materials Company. The average value of molecular weight of PAA was 60000‐80000, and the solvents of PAA were N,N‐dimethylacetamide (DMAC) and N‐methylpyrrolidone (NMP). The chemical structure of PAA was shown in Figure S1g (Supporting Information). Prior to the casting process, the PAA solution was transferred to a beaker and PAA was diluted using N‐methylpyrrolidone (NMP), with a volume ratio of NMP to PAA of 1: 3. Subsequently, the MXene particles were stirred for more than half an hour using a physical stirrer to evenly distribute the MXene particles in the PAA solution in order to prepare different concentrations of MXene/PAA solution (0.3, 0.6, and 0.9 mmol). Subsequently, a specific volume of the mixture was evenly dispensed onto a clean Si wafer using a pipette. To ensure a consistent thickness of the PI‐MXene film, it was annealed on a hotplate to eliminate the solvent and modify the film morphology. The curing process involved sequential treatments at various temperatures: 80 °C for 20 min, 120 °C for 20 min, 160 °C for 30 min, 180 °C for 20 min, 200 °C for 20 min, 220 °C for 20 min, 250 °C for 20 min, and a final stage at 280 °C for 10 min. The PI films with MXene distribution prepared by this annealing process have better mechanical properties. During the curing process, solvents were removed from the PAA at temperatures below 180 °C. Above 180 °C, the anhydrides and amine groups in the PAA undergo a dehydration cyclization reaction to form polyimide chains that were rigid and heat resistant. After curing, the PAA/MXene solution becomes a brown PI film with MXene blend.

### Material Characterization

The surface morphology of **LIMG** was investigated using a high‐resolution field‐emission scanning electron microscope (GeminiSEM360, Zeiss, Germany). The phase and lattice information of the material was studied using a transmission electron microscope (FEI Talos F200X G2, United States). Raman spectra were obtained using a Horiba LabRAM HR Evolution instrument (with a 532 nm laser wavelength) from Japan. X‐ray photoelectron spectroscopy (XPS) measurements were performed on a Thermo Scientific K‐Alpha instrument from the United States, with the spectral lines fitted using Avantage software. The X‐ray diffraction (XRD) instrument used was a Rigaku Ultima IV from Japan with a wavelength of 1.5418 nm, operating at 40 kV and 40 mA.

### Sensor Fabrication

The PI‐MXene film (120 µm thick) was affixed to a glass slide using adhesive tape. The required processing pattern was then input into the software. Subsequently, the material was subjected to direct laser writing with a femtosecond laser (Model: YF‐FL‐20‐100‐1R, from Hangzhou, China) operating at a wavelength of 1030 nm, using an objective of 2X, power of 2000 mW power, 500 kHz repetition rate, and scanning speed of 5 mm s^−1^. Subsequently, a mixture of a polydimethylsiloxane (PDMS) prepolymer and a curing agent (Sylgard 184) in a 10:1 weight ratio was used to encapsulate the sensor surface. The encapsulated sensor surface was cured at 65 °C for 3 h. Silver paste was then applied to the sensor lead positions and wrapped with conductive copper tape to reduce contact resistance. Finally, the extended Cu wires were connected to an electrochemical workstation and other testing source meters.

### Electrical Conductivity Measurements

**LIMG** samples (each measuring 1 × 1 cm) were prepared under varying processing conditions and analyzed for electrical conductivity using a four‐probe conductivity tester model ST2258C from Suzhou Jingge Electronics Co., Ltd. Each sample was tested at four different positions, with the conductivity data presented in the text being an average value from these four positions. Carrier density and carrier mobility were measured using a Hall effect tester model HMS‐7000.

### Electrochemical Properties of LIMG‐sssBased On‐Chip Three‐Electrode System

Potassium ferrocyanide K_4_[Fe(CN)_6_], potassium ferricyanide K_3_[Fe(CN)_6_], potassium chloride (KCl), dopamine hydrochloride (DA, (HO)_2_C_6_H_3_CH_2_CH_2_NH_2_·HCl), uric acid (UA, C_5_H_4_N_4_O_3_), L‐ascorbic acid (AA, C_6_H_8_O_6_) were purchased from Sigma Aldrich. PBS buffer(1 ×) purchased from Aladdin. The characteristics of the three‐electrode system were studied using an electrochemical workstation (CHI660E, Shanghai, China) via cyclic voltammetry (CV) and differential pulse voltammetry (DPV) tests.

### Testing of Pressure and Bending Sensors

The applied pressure was controlled using a universal testing machine (Shimadzu, model AGS‐X), with the bending parameters controlled using a stepper motor. The real‐time resistance data of the sensor were recorded using a GSM‐20H10 digital source meter.

## Conflict of Interest

The authors declare no conflict of interest.

## Supporting information

Supporting Information

## Data Availability

The data that support the findings of this study are available from the corresponding author upon reasonable request.
