# Supplementary material for: High‐Performance Sensing Platform Based on Morphology/Lattice Collaborative Control of Femtosecond‐Laser‐Induced MXene‐Composited Graphene
Source: Adv Sci (Weinh). 2024 Jul 23;11(36):2404889. doi: 10.1002/advs.202404889 (PMC11423250; doi:10.1002/advs.202404889)
Supplement: Supplementary file 1 — Supporting Information [file ADVS-11-2404889-s001.docx]

Supporting Information

Title: High-performance sensing platform based on morphology/lattice collaborative control of femtosecond-laser-induced MXene-composited graphene

*Ruige Su, Misheng Liang^*^, Yongjiu Yuan,Chaojun Huang, Wenqiang Xing, Xiaomeng Bian , Yiling Lian, Bo Wang^*^, Zheng You, and Rui You^*^*

R. Su, M. Liang, C. Huang, W. Xing, X. Bian, R. You

Laboratory of the Intelligent Microsystem

Beijing Information Science and Technology University

Beijing 100192, P.R. China

E-mail: [misheng@bistu.edu.cn](mailto:misheng@bistu.edu.cn);[yourui@bistu.edu.cn](mailto:yourui@bistu.edu.cn)

R. Su, M. Liang, C. Huang, W. Xing, X. Bian, R. You

School of Instrument Science and Opto-Electronics Engineering

Beijing Information Science and Technology University

Beijing 100192, P.R. China

Y. Yuan

Department of Mechanical Engineering

City University of Hong Kong

Hong Kong, 999077, P.R. China

Y. Lian

School of Mechanical Engineering

Beijing Institute of Technology

Beijing 100081, P. R. China

B. Wang

Institute of Medical Equipment Science and Engineering

Huazhong University of Science and Technology

Wuhan, 430074, P. R. China

E-mail: [bow@hust.edu.cn](mailto:bow@hust.edu.cn)

Z. You

State Key Laboratory of Precision Testing Technology and Instruments

Tsinghua University

Beijing, 100084, P. R. China


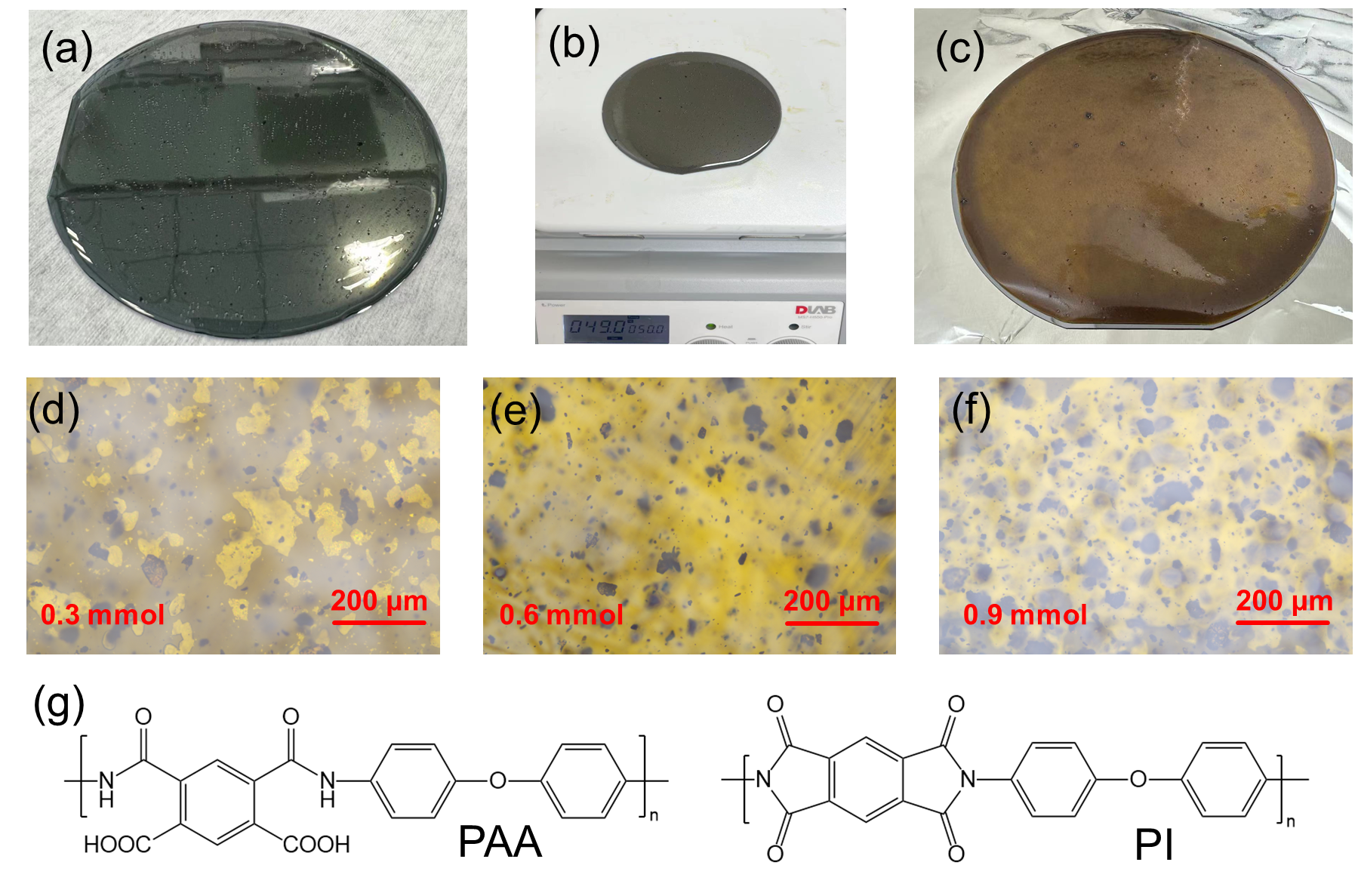


**Figure S1.** Schematic diagram of the fabrication process of polyimide (PI) films doped with varying concentrations of MXene. a) Mixture of poly(amido amine) (PAA) and MXene spread on a silicon wafer. b) Gradient thermal annealing is employed using a hot plate. c) MXene-doped PI film formed post-annealing. d, e, f) Optical microscope focused on the interior of the doped PI films, with schematic illustrations of the optical microscopy for PI films with different doping concentrations presented. g) Molecular structures of PAA and PI.


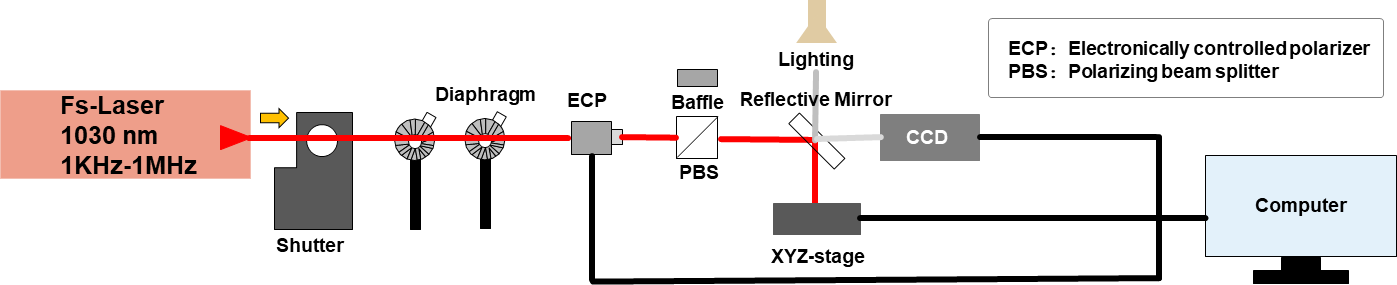


**Figure S2.** Schematic representation of the femtosecond laser direct writing system, comprising a repetition-rate tunable femtosecond laser, an externally controlled polarizer (ECP) system, a CCD imaging system, and an XYZ three-axis translation stage.


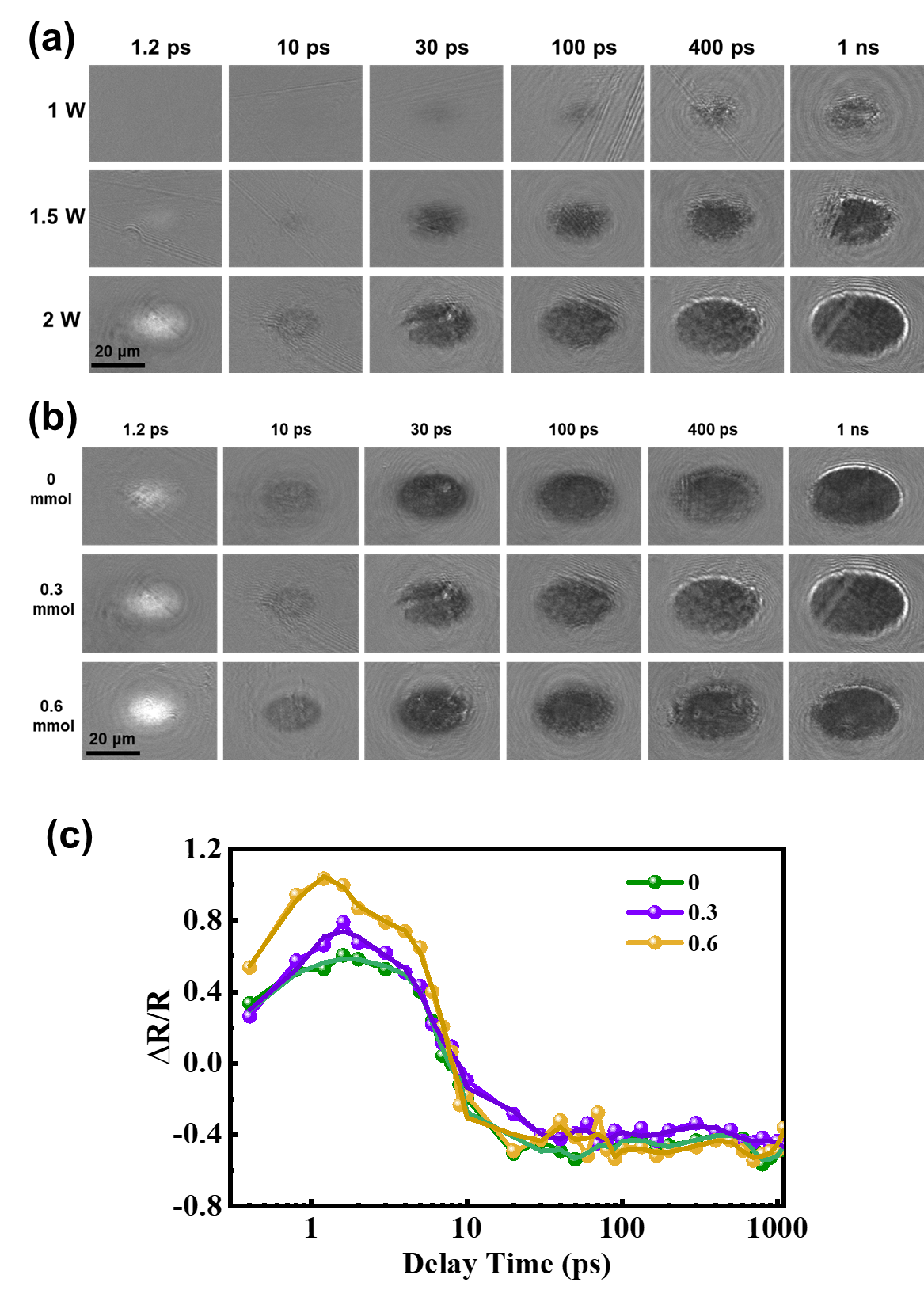


**Figure S3**. a) Pump-probe results of **LIMG** at various power levels (MXene mixing concentration: 0.3 mmol, under the excitation of a fluence ranging from 1 to 2 W). b) Pump-probe results of **LIMG** at different doping concentrations (MXene mixing concentration: 0, 0.3, 0.6 mmol, under the excitation of a fluence ranging from 2 W). c) Variation in the relative reflectivity of **LIMG** at different doping concentrations (MXene mixing concentration: 0, 0.3, 0.6 mmol, under the excitation of a fluence ranging from 2 W).

Figure S3a illustrates the spatiotemporal evolution of the relative reflectance on the surface of PI-MXene under flux excitations ranging from 1 to 2 W. All laser fluxes induce ablation on the material, with surface layers being removed. Notably, higher laser fluxes led to larger irradiated areas. At an irradiance of 1 W, the major axis dimension is less than 10 μm, whereas at 2 W irradiance, the major axis dimension exceeds 20 μm. The size of the molten region also increased with increasing delay time, stabilizing after 400 ps. At 1 W irradiance, no reflectance change was observed initially, with a slight decrease starting at 30 ps, enlarging the ablated region as the delay time increased. However, when the laser flux rises to 1.5 W, the reflectance begins to increase at 1.2 ps, with melting occurring at 10 ps, followed by a continuous decrease in the reflectance and expansion of the molten region. Upon further increasing the laser flux to 2 W, a sharp increase in reflectance was observed at 1.2 ps. At 10 ps, the signal area contracts compared to 1.2 ps, with a sustained decrease in reflectance over longer delays, further expansion of the signal area, and the appearance of a ring-like pattern of alternating light and dark on the edge of the signal area. The central reflectance trend over time extracted from Figure 2a is presented in Figure 2b. The scatter points in Figure 2b are the actual measured changes in relative reflectance; the lines represent the smoothing of the scatter points to facilitate the observation of reflectance trends, allowing for a more detailed description of the ablation process. With an irradiance of 1 W, the relative reflectance begins to decrease after 10 ps, reaching its lowest value at -0.2 after 80 ps, after which the reflectance no longer falls but instead starts fluctuating, possibly owing to mechanical disruption of the surface layer, resulting in fracturing. In this setting, the surface underwent slight melting, accompanied by a weaker mechanical impact.

At an irradiance of 1.5 W, the reflectance initially increased within 1 ps, then decreased, reaching a level below zero at 10 ps and dropping to a low of -0.5 at 30 ps, maintaining approximately -0.5 thereafter. The early rise in reflectance is likely due to the Coulomb explosion, which increases the density of free electrons and surface reflectivity. Subsequently, energy transfer from the electrons to the lattice induces lattice melting, leading to the disappearance of surface fracturing and a more pronounced thermal effect. Intense thermal expansion induces internal thermal stress. When the fluence was increased to 2 W, the density of free electrons dramatically increased to approximately 1 ps, with the peak relative reflectivity reaching 0.8 before declining, nearing zero at 10 ps, eventually resulting in a melting process similar to that observed at 1.54 J/cm^2^. Despite inducing higher electron temperatures and densities compared to that observed at 2 W, the characteristic melting time remains approximately the same, attributed to the inherent frequency of atomic oscillations during the material melting process, which requires atoms to leave their equilibrium positions for melting to occur, thereby limiting the melting rate. The annular pattern observed over a longer timescale may have resulted from material removal and the formation of structures, causing diffraction of the probing light at the edges of the ablation pit.

While distinguishing the differences in the material removal process based on changes in surface reflectivity is challenging, the plasma emission intensity captured by the ICCD at a delay of 5 ns (Figure 2c) shows significant variations in the instantaneous temperature and thermal effects. The plasma intensity at 1.5 W was slightly higher than that at 1 W, and under 2 W irradiation, the plasma ejection intensity was notably greater. Notably, Figure S3b demonstrates that at the same laser fluence, a higher doping concentration resulted in a more pronounced increase in reflectivity between 1–2 ps, indicating a greater increase in the free electron density and enhanced energy absorption, thus leading to more intense ablation. At lower fluences, the vaporization of the material typically results in minimal ablation and a slight reduction in reflectivity, with low levels of introduced defects and oxygen content. However, with increased fluence, thermal effects can cause severe damage to the material by breaking chemical bonds, decomposing the substance, inducing internal strain, and altering its morphology and properties.


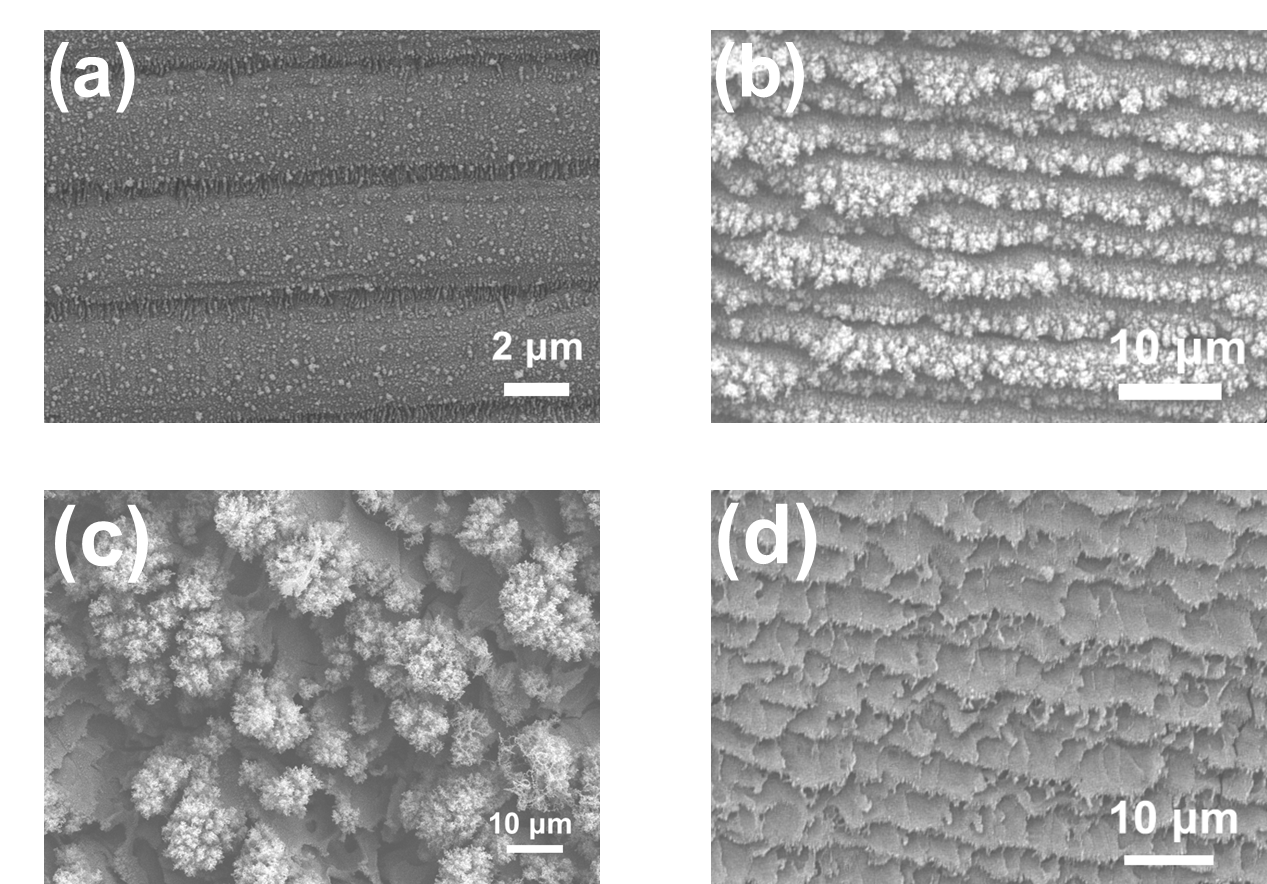


**Figure S4.** SEM images under different power settings at the same scanning speed (5 mm s^-1^), the same MXene mixing concentration (0.6 mmol), and the same laser repetition frequency (500 KHz). a) Laser power is 500 mW; b) Laser power is 1 W. c) Laser power is 1.5 W. d) Laser power is 1.5 W and the area was scanned twice.

**
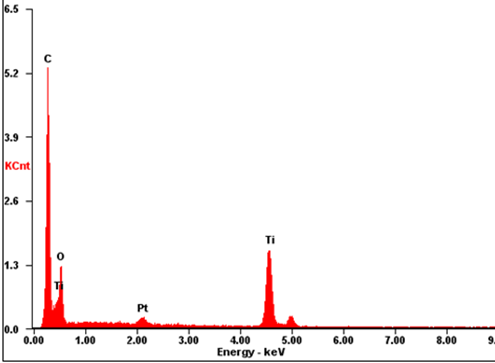
**

**Figure S5.** Elemental analysis spectrograms within the interior of **LIMG** flakes.


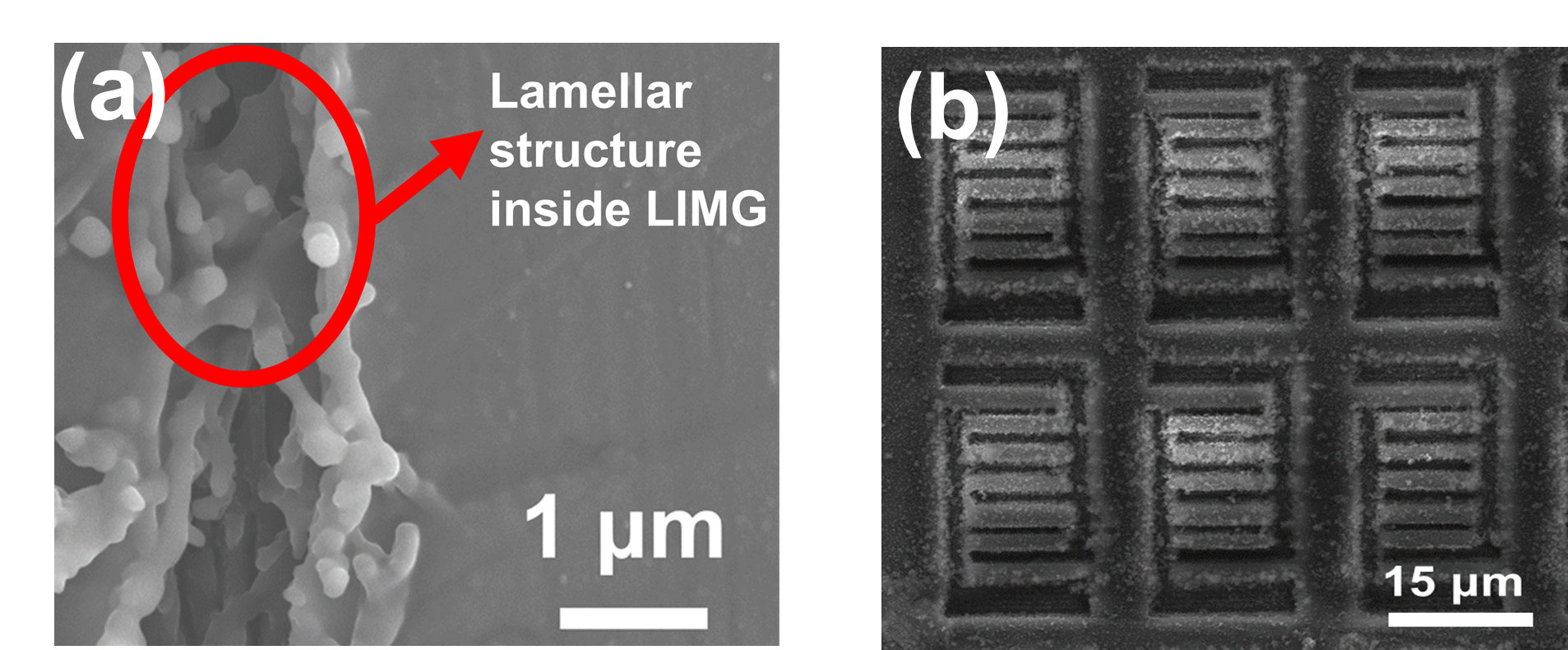


**Figure S6.** a) **LIMG** high-definition SEM images with 1 μm line widths. b) Ultra-micro interdigitated electrodes fabricated on a PI-MXene film using femtosecond laser technology.


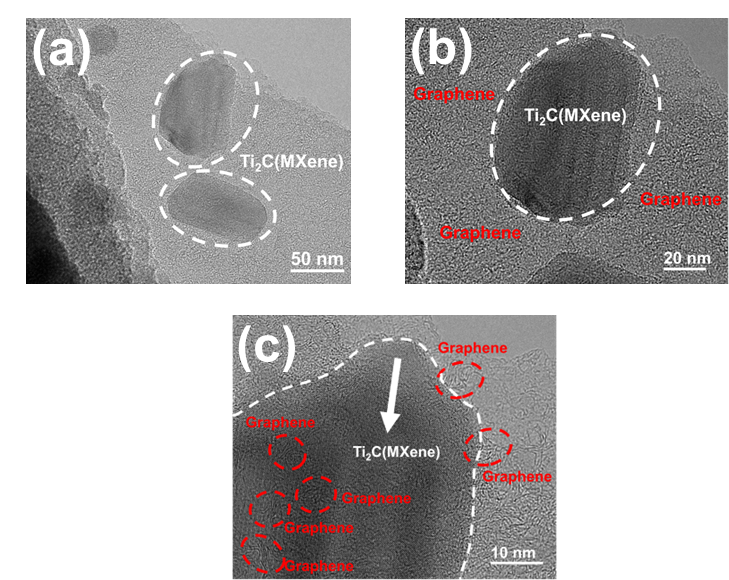


**Figure S7.** a, b, c) Transmission electron microscopy images of **LIMG** at a MXene concentration of 0.6 mmol, a laser repetition frequency of 500 kHz, a laser power of 2W, and a scanning speed of 5 mm s^-1^.

**
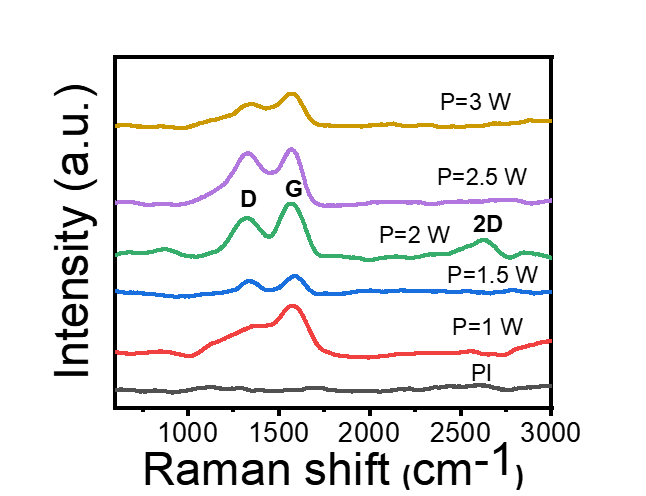
**

**Figure S8.** The Raman spectra of **LIMG** at a processing speed of 5 mm/s, a laser repetition rate of 100 KHz, and an MXene mixture concentration of 0.3 mmol under different laser powers (1–3W).

| Conductivity of **LIMG**（*10^3^ S m^-1^）  Laser Repetition Frequency：**100 KHz** | | | | | | | | | | | |
| --- | --- | --- | --- | --- | --- | --- | --- | --- | --- | --- | --- |
| Doping concentration  of MXene：**0.3 mmol** | | | | Doping concentration  of MXene：**0.6 mmo**l | | | | Doping concentration  of MXene：**0.9 mmol** | | | |
| Power  Speed | 1 W | 1.5 W | 2 W | Power  Speed | 1 W | 1.5 W | 2 W | Power  Speed | 1 W | 1.5 W | 2 W |
| 5 mm/s | 0.714 | 1.351 | 1.369 | 5 mm/s | 1.111 | 2.403 | 1.694 | 5 mm/s | 2.564 | 2.601 | 2.439 |
| 10 mm/s | 0.178 | 1.149 | 0.909 | 10 mm/s | 0.909 | 1.470 | 1.515 | 10 mm/s | 1.639 | 1.428 | 1.851 |
| 15 mm/s | 0.212 | 0.781 | 0.769 | 15 mm/s | 0.714 | 1.464 | 1.002 | 15 mm/s | 1.219 | 1.052 | 1.612 |

**Table S1.** Electrical conductivity test results of the **LIMG** obtained under different MXene doping concentrations, machining speeds, and laser powers, with the laser repetition frequency set at **100 kHz**.

| Conductivity of **LIMG** （*10^3^ S m^-1^）  Laser Repetition Frequency：**500 KHz** | | | | | | | | | | | |
| --- | --- | --- | --- | --- | --- | --- | --- | --- | --- | --- | --- |
| Doping concentration  of MXene：**0.3 mmol** | | | | Doping concentration  of MXene：**0.6 mmo**l | | | | Doping concentration  of MXene：**0.9 mmol** | | | |
| Power  Speed | 1 W | 1.5 W | 2 W | Power  Speed | 1 W | 1.5 W | 2 W | Power  Speed | 1 W | 1.5 W | 2 W |
| 5 mm/s | 0.833 | 1.204 | 2.561 | 5 mm/s | 1.998 | 2.325 | 3.187 | 5 mm/s | 0.625 | 2.631 | 2.325 |
| 10 mm/s | 0.556 | 1.415 | 1.639 | 10 mm/s | 1.401 | 1.945 | 2.381 | 10 mm/s | 0.271 | 0.833 | 0.123 |
| 15 mm/s | 0.437 | 0.588 | 1.219 | 15 mm/s | 1.123 | 1.625 | / | 15 mm/s | 0.252 | 0.769 | 0.943 |

**Table S2.** Electrical conductivity test results of the **LIMG** obtained under different MXene doping concentrations, machining speeds, and laser powers, with the laser repetition frequency set at **500 kHz**.

| Conductivity of **LIMG** （*10^3^ S m^-1^）  Laser Repetition Frequency：**1 MHz** | | | | | | | | | | | |
| --- | --- | --- | --- | --- | --- | --- | --- | --- | --- | --- | --- |
| Doping concentration  of MXene：**0.3 mmol** | | | | Doping concentration  of MXene：**0.6 mmo**l | | | | Doping concentration  of MXene：**0.9 mmol** | | | |
| Power  Speed | 1 W | 1.5 W | 2 W | Power  Speed | 1 W | 1.5 W | 2 W | Power  Speed | 1 W | 1.5 W | 2 W |
| 5 mm/s | 0.370 | 2.04 | 1.388 | 5 mm/s | 1.162 | 1.851 | 2.506 | 5 mm/s | 0.833 | 2.041 | 2.631 |
| 10 mm/s | 0.294 | 0.123 | 0.769 | 10 mm/s | 0.873 | 1.639 | 1.961 | 10 mm/s | 0.385 | 1.315 | 1.785 |
| 15 mm/s | 0.181 | 0.124 | 0.667 | 15 mm/s | 0.714 | 1.470 | 1.449 | 15 mm/s | 0.217 | 1.563 | 1.587 |

**Table S3.** Electrical conductivity test results of the **LIMG** obtained under different MXene doping concentrations, machining speeds, and laser powers, with the laser repetition frequency set at **1 MHz**.


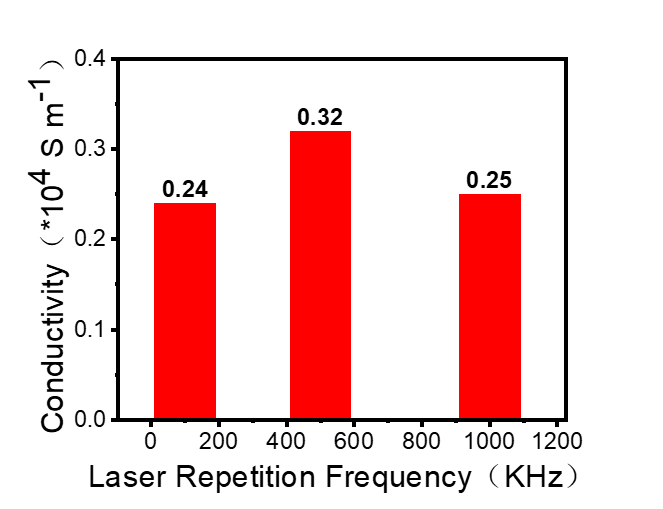


**Figure S9.** Electrical conductivity of **LIMG** at various laser repetition rates.


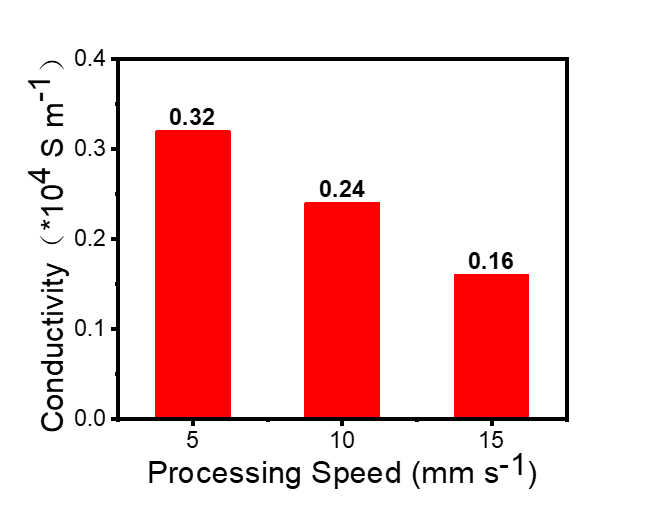


**Figure S10.** Electrical conductivity of **LIMG** at different laser scanning speeds.


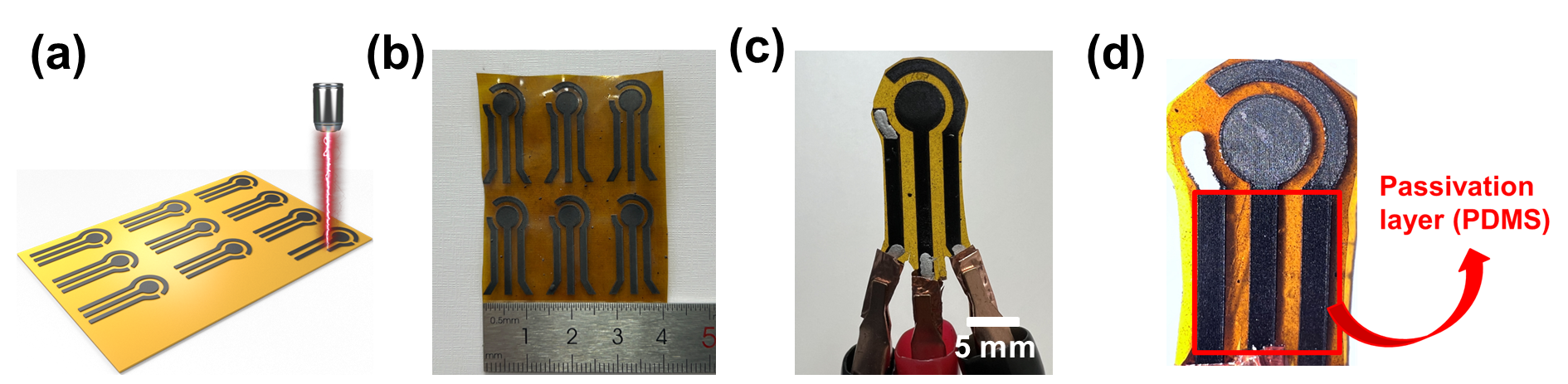


**Figure S11.** a) Schematic illustration of electrochemical chips mass-produced via in-situ patterning with femtosecond laser technology. b) Physical photo of the **LIMG**-based electrochemical chip. c) Application of silver paste at the reference electrode position. At the electrode tail interface, silver paste is applied to eliminate contact resistance and lead out copper wires, with copper foil tape utilized to secure the copper wires in place. d) Non-working areas of the electrochemical chip are passivated with PDMS coverage (the area in the red box appears darker due to the PDMS overlay).


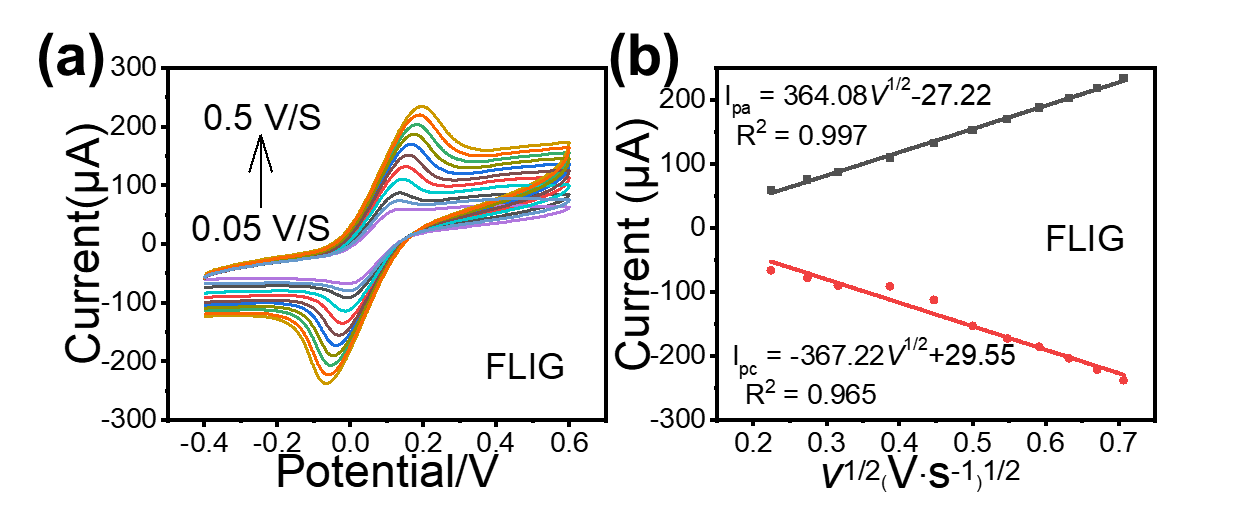


**Figure S12.** a) Cyclic voltammetry (CV) measurements conducted at different scan rates (0.05, 0.75, 0.1, 0.15, 0.2, 0.25, 0.3, 0.35, 0.4, 0.45, and 0.5 V·s^−1^) of the FLIG-based electrode in 5 mM K_3_[Fe(CN)_6_]/K_4_[Fe(CN)_6_] and 0.1 M KCl. d) Peak current plotted as a function of the square root of the scan rate with fitted linear regression curves in 5 mM K_3_[Fe(CN)_6_]/K_4_[Fe(CN)_6_] and 0.1 M KCl. Black and red dots indicate anodic and cathodic peak currents, respectively.


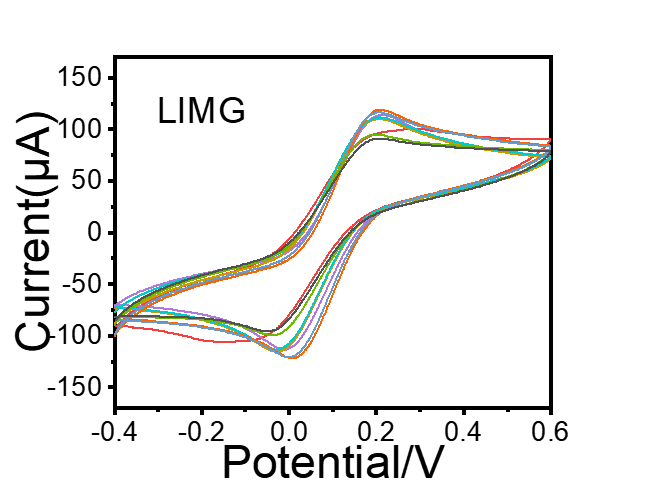


**Figure S13.** CV curves of eight different electrochemical sensors fabricated using identical processing parameters in 5 mM K_3_[Fe(CN)_6_]/K_4_[Fe(CN)_6_] and 0.1 M KCl.


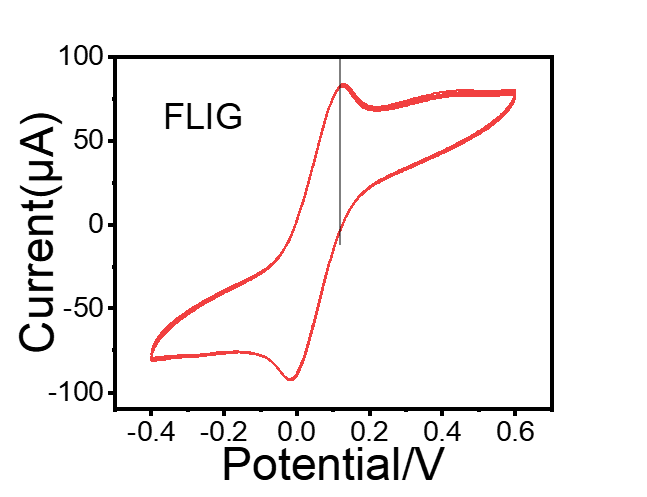


**Figure S14.** The CV of 10 cycles of FLIG was recorded at a scan rate of 0.1 V·s^-1^ in 5 mM K_3_[Fe(CN)_6_]/K_4_[Fe(CN)_6_] and 0.1 M KCl.


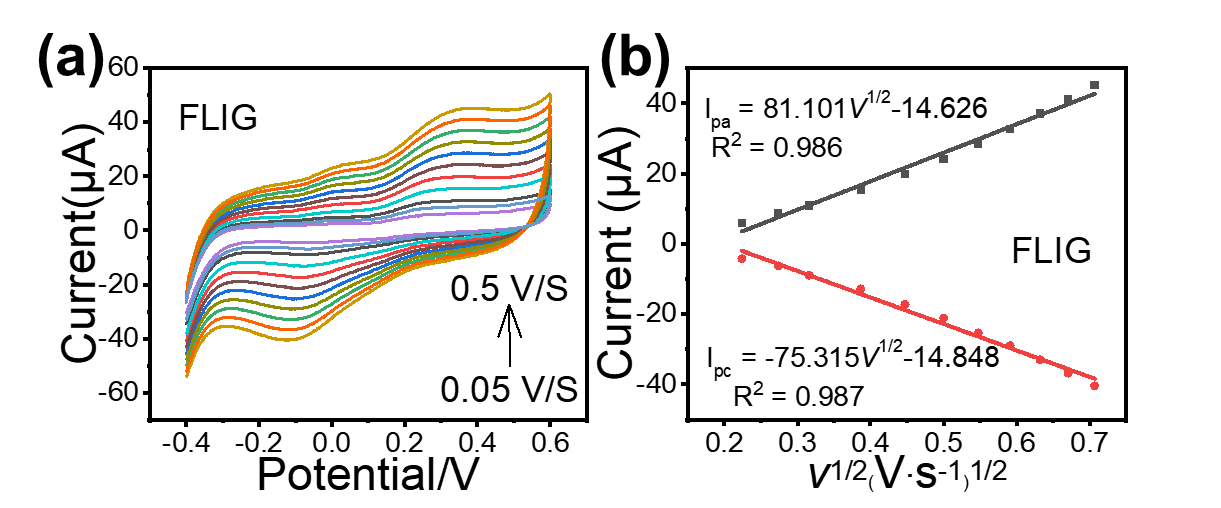


**Figure S15.** a) CV measurements conducted at different scan rates (0.05, 0.75, 0.1, 0.15, 0.2, 0.25, 0.3, 0.35, 0.4, 0.45, and 0.5 V·s^−1^) of the FLIG-based electrode in 100 μM UA and 0.01 M PBS. b) Peak current plotted as a function of the square root of the scan rate with fitted linear regression curves in 100 μM UA and 0.01 M PBS. Black and red dots indicate anodic and cathodic peak currents, respectively.


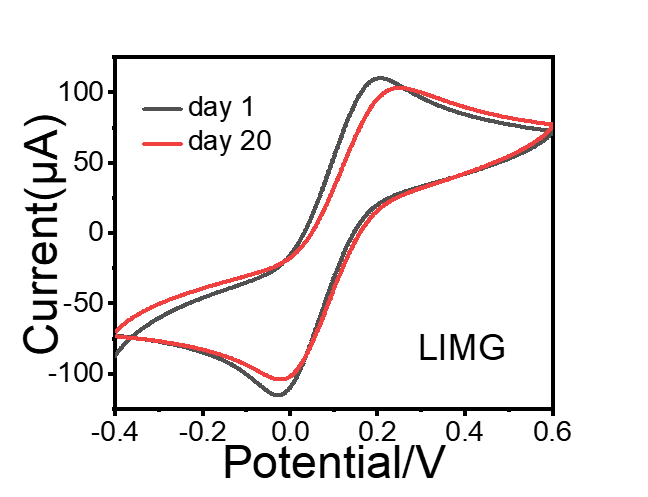


**Figure S16.** Comparison of peak CV current in 5 mM K_3_[Fe(CN)_6_]/K_4_[Fe(CN)_6_] and 0.1 M KCl for **LIMG**-based electrochemical sensors at a sweep rate of 0.1 V·s^-1^ on day 1 and day 20.


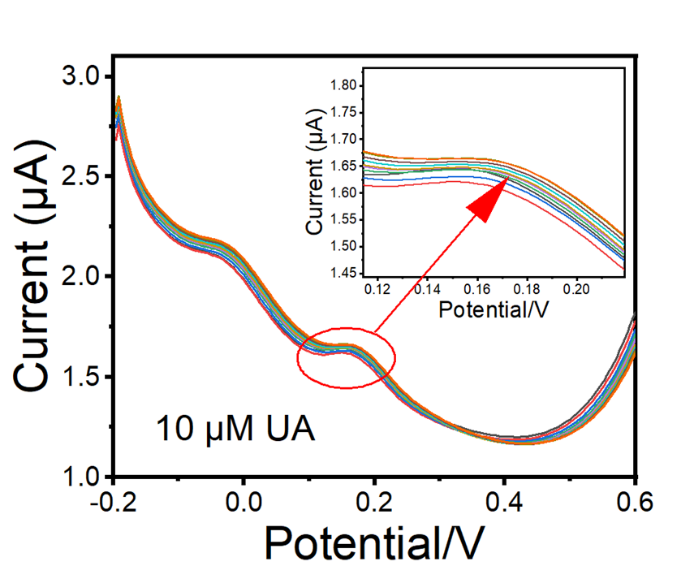


**Figure S17.** At a UA concentration of 10 μM, the standard deviation was calculated to be 1.40099 × 10^-8^, per the response curves obtained through ten scans of DPV.


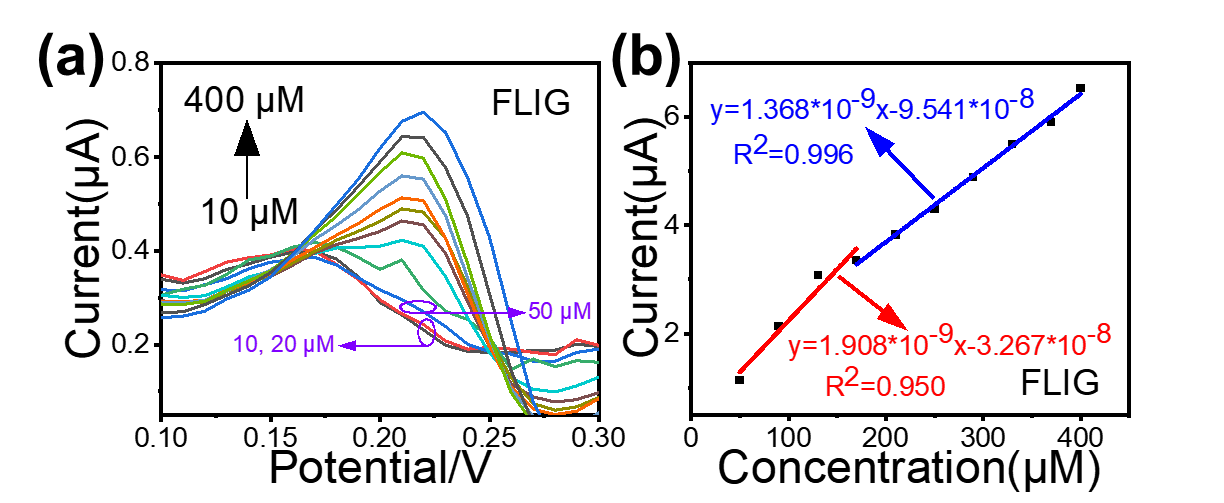


**Figure S18.** a) DPV curves measured with different concentrations of UA using the FLIG-based electrode. b) Fitted calibration curve of the FLIG-based electrode between peak currents and UA concentrations.


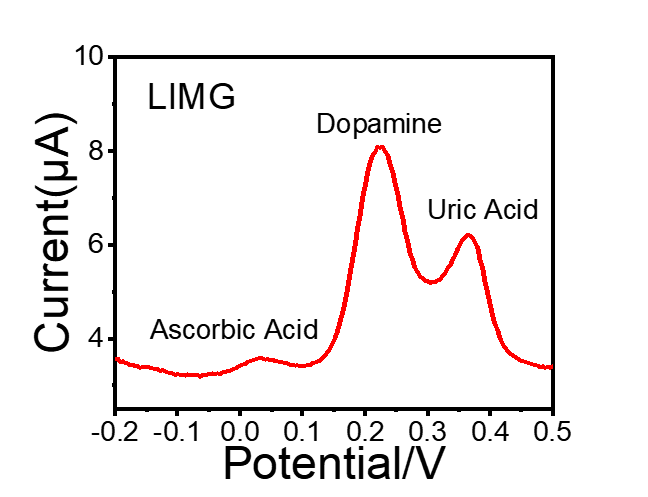


**Figure S19.** Selectivity testing of **LIMG**-based electrochemical sensors in a mixed PBS solution of 100 μM AA , 100 μM DA and 100 μM UA.


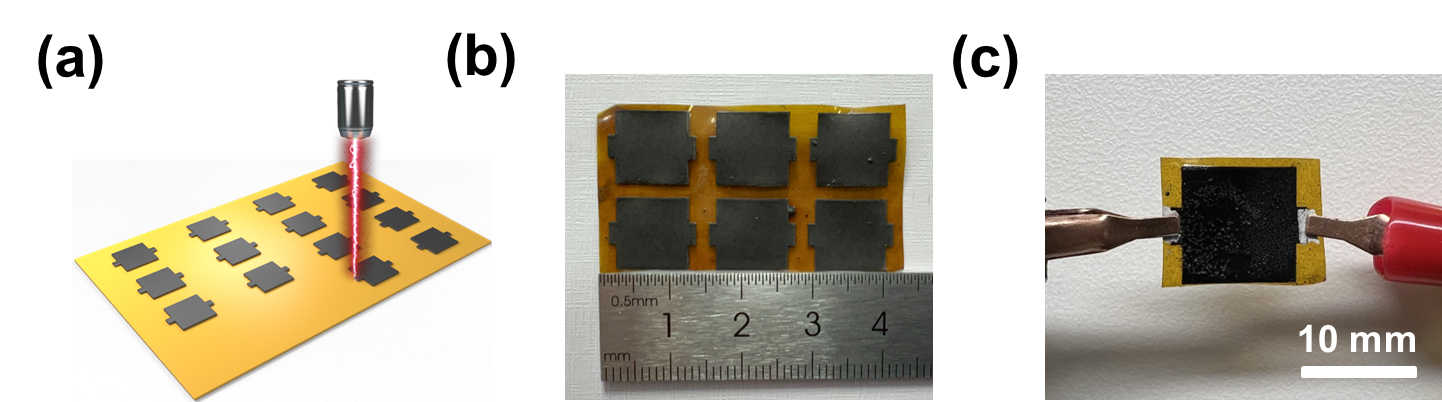


**Figure S20.** a) Schematic illustration of pressure sensors mass-produced via in-situ patterning with femtosecond laser technology. b) Physical photo of the **LIMG**-based pressure sensor. c) Silver paste is applied to the interfaces at both ends of the pressure sensor, followed by leading-out copper wires. Subsequently, a PDMS encapsulation is applied to the surface layer, facilitating testing and application.


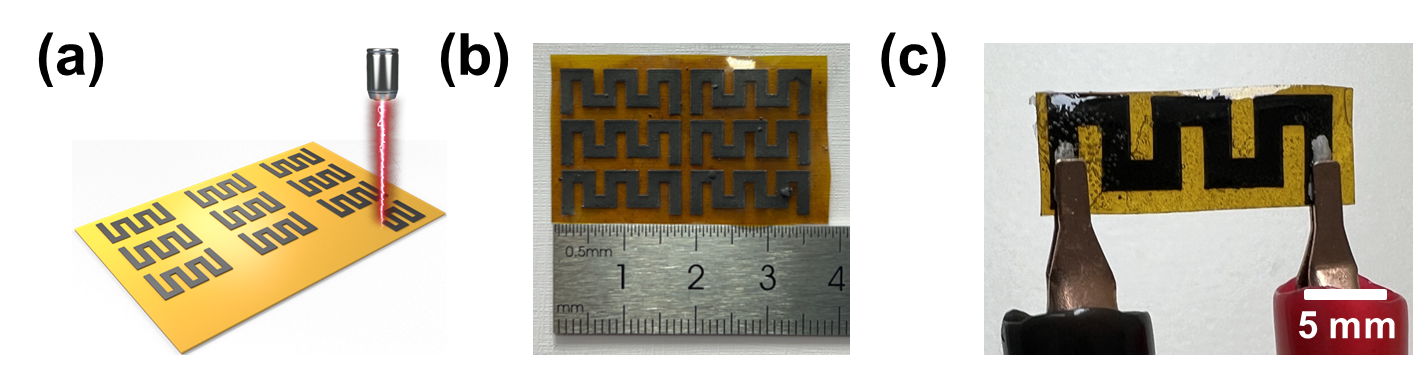


**Figure S21.** a) Schematic diagram of the snake-shaped bending sensor mass-produced through in-situ patterning by femtosecond laser. b) Physical photo of the **LIMG**-based snake-shaped bending sensor. c) Silver paste is applied to the interfaces at both ends of the snake-shaped bending sensor, followed by leading-out copper wires, with the surface encapsulated with PDMS.


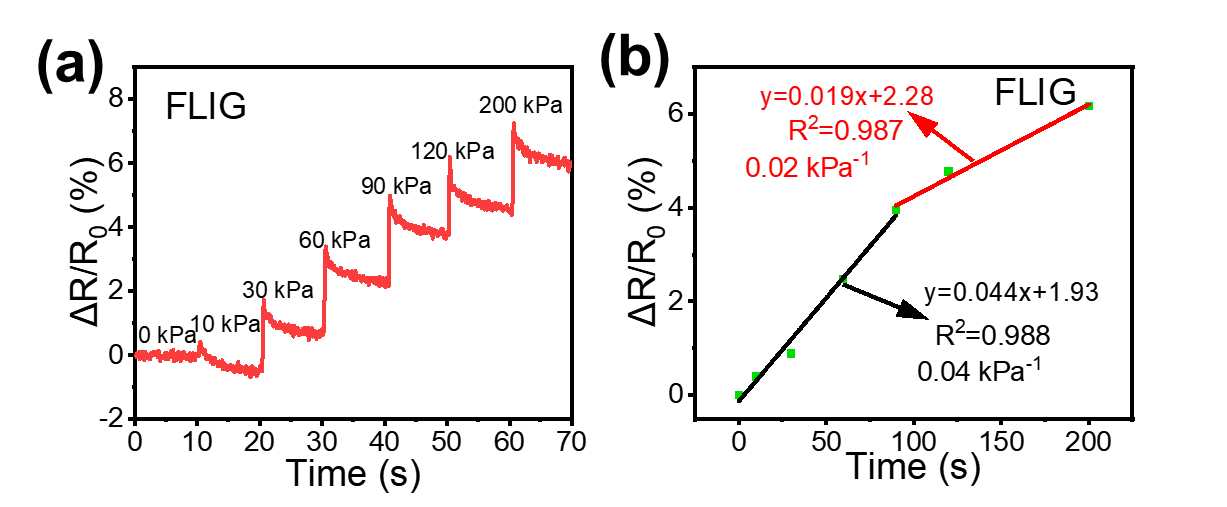


**Figure S22.** a) Relative change in resistance of the FLIG during progressive pressurization. b) Relative change in resistance of the pressure sensor of the FLIG under external pressure and its sensitivity.

| Modified electrode | Laser | Response range (kPa) | Sensitivity (kPa^-1^) | Reference |  |
| --- | --- | --- | --- | --- | --- |
| **LIMG/PDMS** | **Fs-Laser (1030 nm)** | **0-100 kPa**  **100-200 kPa** | **1.5*10^-1^**  **7*10^-2^** | **This work** | |
| PDMS/LIG/Fabric | CO_2_ laser | 0-30 kPa  30-220 kPa | 1.2*10^−3^  8.5*10^−3^ | ^[1]^ | |
| LIG | Nd:YVO_4_ laser | 0-1132 kPa | 2*10^-2^ | ^[2]^ | |
| LIG | Semiconductor laser（915 nm） | 0-65 kPa | 1.68*10^-1^-4.5*10^-2^ | ^[3]^ | |
| LIG/GaInSn | CO_2_ laser | 0-80 kPa  80-140 kPa | 2.73*10^-2^  1.049*10^-1^ | ^[4]^ | |
| LIG/PDMS | CO_2_ laser | 0-400 kPa | 8.8*10^-1^-6.2*10^-3^ | ^[5]^ | |

**Table S4.** The performance of the single-layer piezoresistive pressure sensor based on **LIMG** was compared with other reported sensors based on LIG.

| Modified electrode | Manufacturing method | LOD（μM） | Reference |
| --- | --- | --- | --- |
| **LIMG** | **Femtosecond laser induction** | **2.48** | **This work** |
| LIG/BSA and Tween-20 | Laser induction/Spin coating | 2.1 | ^[6]^ |
| GO/ZnO-NF | Hydrothermal | 5.4 | ^[7]^ |
| Paper-LIG | Laser induction | 3.97 | ^[8]^ |
| Graphene/Polydopamine/  Carbon nanotubes | Hydrothermal | 15 | ^[9]^ |
| LIG/Au/Pd/MXene | Laser induction/self-assembly | 1.47 | ^[10]^ |
| rGO/AuNPs | Electrodeposition | 3.6 | ^[11]^ |
| rGO | Laser restoration | 9.67 | ^[12]^ |
| LIG/ Iron nano-catalysts | Laser induction/ Drip | 1.37 | ^[13]^ |
| rGO | Hydrothermal | 27 | ^[14]^ |
| WO_3_ /MoO_3_ /MoS_2_ /rGO | Hydrothermal | 2.4 | ^[15]^ |

**Table S5.** The performance of the **LIMG**-based UA sensor was compared with other reported graphene composite-based sensors.

**References:**

[1] T. Raza, M. K. Tufail, A. Ali, A. Boakye, X. Qi, Y. Ma, A. Ali, L. Qu, M. Tian, *ACS Applied Materials & Interfaces* **2022**, 14, 54170.

[2] A. F. Carvalho, A. J. Fernandes, R. Martins, E. Fortunato, F. M. Costa, *Advanced Materials Technologies* **2020**, 5, 2000630.

[3] L. Chen, B. Hu, X. Gao, F.-l. Chang, H. Yang, G.-j. He, X.-w. Cao, X.-l. Zou, X.-c. Yin, *Composites Science and Technology* **2022**, 230, 109790.

[4] Y. Li, G. Matsumura, Y. Xuan, S. Honda, K. Takei, *Advanced Functional Materials* **2024**, 2313824.

[5] M. Sun, S. Cui, Z. Wang, H. Luo, H. Yang, X. Ouyang, K. Xu, *Microsystems & Nanoengineering* **2024**, 10, 50.

[6] J. Nong, N. Zhang, A. Wen, C. Hu, *Journal of Electroanalytical Chemistry* **2024**, 952, 117982.

[7] S. Tariq, U. Saeed, S. Riaz, A. Saqib, S. Khurshid, M. H. Nawaz, *Materials Today Communications* **2024**, 39, 108902.

[8] B. Kulyk, S. O. Pereira, A. J. Fernandes, E. Fortunato, F. M. Costa, N. F. Santos, *Carbon* **2022**, 197, 253.

[9] C. Wang, J. Li, K. Shi, Q. Wang, X. Zhao, Z. Xiong, X. Zou, Y. Wang, *Journal of electroanalytical Chemistry* **2016**, 770, 56.

[10] Y. Wang, P. Zhao, B. Gao, M. Yuan, J. Yu, Z. Wang, X. Chen, *Microchemical Journal* **2023**, 185, 108177.

[11] F. Mazzara, B. Patella, G. Aiello, A. O'Riordan, C. Torino, A. Vilasi, R. Inguanta, *Electrochimica Acta* **2021**, 388, 138652.

[12] R. K. Singh, P. S. Kumar, K. Amreen, S. K. Dubey, S. Goel, *IEEE Transactions on NanoBioscience* **2022**, 22, 548.

[13] P. Zhao, Y. Zhang, Y. Liu, D. Huo, J. Hou, C. Hou, *Biosensors and Bioelectronics* **2024**, 249, 116012.

[14] F. Besbes, Z. Hsine, R. Mlika, *Carbon Letters* **2023**, 33, 2109.

[15] T. S. I. Bakavaty, K. Gurunathan, *Materials Science and Engineering: B* **2024**, 299, 116967.
